# 3D-printed vancomycin-eluting PGCL/MXene bifunctional scaffold for management of infected bone defects

**DOI:** 10.1016/j.mtbio.2025.101847

**Published:** 2025-05-07

**Authors:** Xipeng Chen, Yuanpei Cheng, Yongbo Li, Ze Tan, Han Wu

**Affiliations:** a Department of Orthopedics, China-Japan Union Hospital of Jilin University, Changchun, 130033, China; b Shandong First Medical University Affiliated Provincial Hospital, Jinan, 250100, China

**Keywords:** Ti_3_C_2_, Vancomycin, Bone regeneration

## Abstract

The clinical management of infected bone defects remains a significant challenge in orthopedic surgery. In this study, we developed a multifunctional composite scaffold by integrating MXene (Ti_3_C_2_) nanosheets and vancomycin (Van) into a degradable poly(glycolide-co-caprolactone) (PGCL) polymer through precision 3D printing technology, with the aim of regenerating acutely infected bone defects. Systematic optimization established the optimal composition as 5 wt% Ti_3_C_2_ and 5 wt% Van within the degradable PGCL polymer. Compared with PGCL scaffolds, a significantly thicker mineralized layer could be deposited on the PGCL@5 %Ti_3_C_2_/5 %Van surface in simulated body fluid (SPF). The *in vitro* results showed that the PGCL@5 %Ti_3_C_2_/5 %Van scaffold had antibacterial properties, cytocompatibility, osteoblast differentiation and extracellular mineralization. The *in vivo* results showed that the PGCL@5 %Van/5 %Ti_3_C_2_ scaffold effectively inhibited infection caused by methicillin-resistant *Staphylococcus aureus (MRSA)* in bone defects and promoted bone repair. The potential mechanism of Ti_3_C_2_ in anti-inflammatory and osteogenic induction was investigated through transcriptome analysis. These results suggest that Ti_3_C_2_ is involved in the regulation of the PI3K‒Akt signalling pathway to increase osteogenic induction ability and regulate the inflammatory response by downregulating the NF-κB pathway through the deiodinase iodothyronine type II (DiO2). These findings indicate that the PGCL@5 %Ti_3_C_2_/5 %Van scaffold can enhance the regeneration of acutely infected bone defects.

## Graphical abstract

**Figure:**
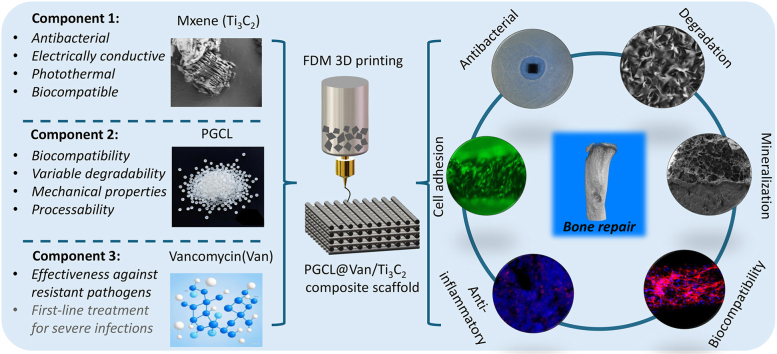

## 1 Introduction

Open fractures, severe soft tissue injuries, diabetes and other factors are the main causes of infected bone defects [[1], [2], [3]]. Local infection can exacerbate tissue damage and destroy the capacity for bone regeneration, increasing the difficulty of bone repair [4]. Although many factors are associated with bone infection, methicillin-resistant *Staphylococcus aureus (MRSA)* remains the most prevalent and destructive pathogen and is a great challenge in clinical treatment in particular [5]. Traditional standard treatments for infectious bone defects include two main missions, namely, infection control and reconstruction of bone defects [6,7]. Systemic administration of antibiotics is a common anti-infective strategy. However, it is difficult to achieve effective drug concentrations at the lesion site because of the complex infection environment and local destruction of the vasculature and bone [8]. Although antibiotic-impregnated spacers can deliver antibiotics topically, secondary surgery is needed approximately 6 weeks after the first operation to remove spacers [9,10]. The repair of bone defects with bone grafting commences only after the infection process is controlled. A treatment platform with both antibacterial and osteogenic functions can simplify the therapeutic process through one implantation procedure. The irregular defect site needs to be filled and fixed with suitable materials to restore the original shape and function.

Materials or drugs that promote osteogenesis are combined with antibiotics or antibacterial materials to develop several engineered scaffolds with dual functions [11,12]. Many natural polymers and synthetic polymers with biocompatibility and biodegradability are used as substrate components for engineered scaffold development. Most natural polymers, such as chitosan, collagen, gelatin, and hyaluronic acid, are derived from extracellular matrix (ECM) proteins. They are characterized by inherent biological activity, biodegradability, natural remodelling, and hydrophilicity. However, the potential disadvantages are fast biodegradation and weak mechanical strength [13]. Biodegradable synthetic polymers are excellent biomedical materials. The physical and chemical properties of these polymers can be designed at the molecular level to control their mechanical and degradation properties for ideal applications. Many polymers, such as polylactic acid (PLA), poly(lactic-co-glycolic acid) (PLGA), polyurethane and poly(ε-caprolactone) (PCL) have been applied to encapsulate antibacterial components [14]. Compared with other materials, poly(glycolide-co-caprolactone) (PGCL) can be easily fabricated into complex structures due to its high plasticity and is widely used in additive manufacturing, including 3D printing and rapid prototyping, to meet the 3D shape requirements of bone substitutes at defect locations [15]. Its elastic strength and degradation rate can be regulated by adjusting the ratio of glycolide (GA) to caprolactone (CL), making it a good candidate drug carrier [16]. However, since PGCL is a bioinert polymer material, it is necessary to combine PGCL with osteogenic components to increase its osteogenic activity.

Recently, two-dimensional (2D) nanomaterials have shown attractive applications in catalysis, optical/electronic devices, and biomedicine due to their favourable physicochemical properties, such as large specific surface areas, ultrathin 2D structures, photothermal capabilities, photocatalytic properties, electrical conductivity, and biocompatibility [17,18]. As novel 2D nanomaterials, MXenes have demonstrated antibacterial, immunomodulatory and anticancer properties [[19], [20], [21], [22], [23]]. In addition, MXenes also exhibit osteogenic activity. Zhang et al. fabricated multilayer MXene films and investigated their osteogenic activity in MC-3T3 cells [24]. The results showed that the MXene films were biocompatible and promoted the osteogenic differentiation of MC3T3 cells. The mRNA expression levels of ALP, OCN and OPN in MC3T3 cells were significantly increased. In a rat calvarial defect model, MXene films were also observed to bind well to tissue and promote new bone formation. Awasthi et al. fabricated PCL-MXene electrospun membranes and evaluated their bioactivity. The results showed that the PCL-MXene electrospun membranes promoted the mineralization of osteoblasts and accelerated the deposition of calcium and phosphorus on the fibre surface [25]. Huang et al. constructed PLLA-MXene composite nanofibers and reported that the composite nanofibers significantly promoted the osteogenic differentiation of BMSCS [26]. The expression levels of collagen 1, OCN, OPN, RUNX2 and other osteogenesis-related proteins in cells were significantly increased. By 3D printing, Mi et al. constructed MXene-incorporated sodium alginate/hydroxyapatite composite scaffolds. The results showed that the scaffold had good biocompatibility and promoted the proliferation of the BMSCs. The expression of ALP and osteogenic genes was upregulated. The formation of mineralized nodules on the composite scaffolds was promoted. It also significantly promoted bone healing in a skull defect model [27]. These studies have proven the potential of MXene materials in bone repair. However, the unclear molecular mechanisms involved in bone repair by MXenes still need to be studied.

In this study, MXene (Ti_3_C_2_) nanosheets and vancomycin (Van) were integrated into the biodegradable polymer PGCL to fabricate a dual-functional composite scaffold *via* 3D printing for the regeneration of acute infected bone defects. The physical and chemical properties of the composite scaffolds were characterized. The anti-infective ability and osteopromoting activity of the composite scaffolds were tested both *in vitro* and *in vivo*. Furthermore, the potential mechanism of MXene materials in the field of inflammation regulation and bone regeneration was explored *via* genomic analysis. These scaffolds provide a new platform for the treatment of infected bone defects and have the potential to obtain regulatory approval for clinical use.

## 2 Materials and methods

### 2.1 Materials

PGCL (MW = 70,000 Da) was purchased from Jinan Dagang Biological Engineering Co., Ltd. Ti_3_C_2_ was purchased from Jilin Yiyi Technology Co., Ltd. Van was purchased from Shanghai Aladdin Biochemical Technology Co., Ltd.

### 2.2 3D-printed scaffolds

To explore the optimal amount of Ti_3_C_2_ and Van in dual-function composite scaffolds, scaffolds with different amounts of Ti_3_C_2_ or Van were fabricated. For the construction of PGCL@Ti_3_C_2_, PGCL particles were melted and mixed with Ti_3_C_2_ powder (200 mesh) *via* an internal mixer (Yiyang Rubber and Plastics Machinery Group Co., Ltd.) at 40 rpm and 100 °C for 10 min. The mass percentages of Ti_3_C_2_ in different proportions of the composites were 1 %, 5 %, and 10 %. For the construction of PGCL@Van, Van was added to melted PGCL and mixed at 100 °C for 10 min. The mass percentages of Van in the different PGCL@Van composites were 1 %, 3 %, 5 % and 10 %. The composite materials with different Ti_3_C_2_ or Van ratios (Table S1) were heated and stirred evenly and printed *via* a fused deposition molding (FDM) 3D printer (3DCreator 02, Ubbiotech, China) to prepare scaffolds. A PGCL scaffold was fabricated as a control. The dimensions of the scaffold were designed using SolidWorks 2017 software and saved in STL format for 3D printing. The printing parameters were set by Simplyfy3D software as follows: the printing speed was 10 mm/s, the temperature of the printing head was 100 °C, and the filling density was 50 %. The optimized scaffolds in the subsequent experiments were all fabricated *via* the same parameters.

### 2.3 Physical and chemical properties of the scaffolds

The appearance of the scaffolds was observed and photographed with a digital camera (Z5, Nikon, Japan). The surface morphology of the composite scaffolds was observed by scanning electron microscopy (SEM, Gemini 2, ZEISS, Germany) at an acceleration voltage of 5 kV. The distributions of C, N, O and Ti on the sample surface were observed *via* energy dispersion spectroscopy (EDS) mapping. Micro-CT (μCT100, SCANCO, Switzerland) was used to scan the scaffolds. The parameters were set as follows: the voltage was 45 kV, the current was 145 μA, and the integration time was 400 ms. A three-dimensional structure of the polymer scaffold was constructed. After scanning, the porosity of the scaffold was calculated *via* the CTAn software program. Fourier transform infrared (FTIR, FTIR-2000, Perkin Elmer, USA) spectral analysis was performed to characterize the chemical compositions of the different materials. The contact angle was measured on a contact angle system (Kruss DSA30, Germany). The different scaffolds were tested *via* a modular compact rheometer (MCR) 302 (Anton Paar GmbH, Germany) to obtain the dynamic (shear) viscosity. The temperature was set at 100 °C, and the shear rate was 1/s. Thermogravimetric analysis (TGA) of each material was performed *via* a thermal analyser (TGA2, METTLER TOLEDO, Switzerland). The samples were tested from 25 °C to 800 °C under a nitrogen atmosphere at a heating rate of 10 °C/min. The mechanical properties of the scaffolds were tested at room temperature *via* a universal mechanical tester (5500, Instron, USA). The indenter displacement rate for measuring the compressive strength of the scaffolds was 2 mm/min, and the fixture displacement rate for measuring the tensile strength was 20 mm/min.

### 2.4 Release efficiency of van from the scaffolds

Van was dissolved in normal saline to prepare a series of standard solutions with gradient concentrations. The absorbance of the standard solution at a wavelength of 280 nm was measured *via* a microplate reader (Tecan M200, Switzerland). A linear relationship curve was generated for the relationship between the concentration of Van solution and the OD value. The Van loaded scaffolds (8 mm × 8 mm × 2 mm) were immersed in 10 ml of PBS (pH 7.4), followed by shaking at 37 °C and 60 rpm/min 1 ml solution was collected at regular intervals, and 1 ml of normal saline was added. The absorbance of the immersion solution at 280 nm was measured *via* the standard linear equation to calculate the concentration of released Van.

### 2.5 In vitro antibacterial ability assay

3D printing of PGCL was performed at 100 °C. Therefore, the effect of high temperature on the antimicrobial activity of Van was tested. The Van were heated at different temperatures for 30 min. *MRSA* was first cultured in sterile Luria Bertani (LB) medium at 37 °C with continuous shaking (100 rpm) until the OD_600_ of the bacterial suspension reached 1.0. After that, 2 mL of the 1000-fold-diluted bacterial suspension was transferred to a 10 ml centrifuge tube. Different amounts of treated Van were then added to the bacterial suspension. After being cultured at 37 °C with continuous shaking (100 rpm) for another 8 h, the OD_600_ of the bacterial suspension was measured. The inhibition zone method was performed according to the Clinical and Laboratory Standards Institute (CLSI). One hundred microliters of *S.aureus* suspension, which was diluted to approximately 1.0 × 10^6^ colony-forming units (CFU)/mL, was spread evenly over LB solid medium. The sterilized scaffolds (8 mm × 8 mm × 2 mm) were aseptically transferred onto LB agar plates. Following 24 h of incubation at 37 °C, the inhibition zones were documented photographically and measured quantitatively. Polymethyl methacrylate (PMMA, Heraeus, Germany) cement was used for comparison with the clinically used products for the inhibition zone experiment.

### 2.6 Degradation properties of the scaffolds

The scaffolds were immersed in 10 ml of PBS (containing 5 % lipase) and placed in a thermostatic oscillator (BE1200, Kylin-Bell, China) at a speed of 60 rpm/min at 37 °C. The weight loss rate of the scaffold was calculated on day 15 after thorough freeze-drying. The surface and cross section of the material were observed *via* SEM (Gemini 2, ZEISS, Germany). The weight loss rate was calculated *via* the following formula (1):

(1) $$\text{Weight}\;\text{loss}\;\text{rate}\;\left(\%\right)=\left(W_{0}‐W_{t}\right)/W0\times 100\%$$

where *W*_*0*_ represents the weight at 0 days, and *W*_*t*_ represents the weight on day 15.

### 2.7 Biomimetic mineralization of the scaffolds

The scaffolds were immersed in 10 ml of simulated body fluid (SBF, Solarbio, China) and placed in a thermostatic oscillator (BE1200, Kylin-Bell, China) at a speed of 60 rpm/min at 37 °C to simulate the dynamic process and avoid uneven mineralization of the scaffolds after standing long. The SBF mixture was exchanged every other day to maintain the microenvironment required for scaffold mineralization. After a 15-day incubation period, the scaffolds were thoroughly rinsed with deionized water and then subjected to freeze-drying. The surface and cross - section of the material were examined *via* SEM. Moreover, the thickness of the mineralized layer was measured using ImageJ software (NIH, US).

### 2.8 Cytocompatibility and immunofluorescence (IF)

MC-3T3-E1 cells were used in this study to evaluate the cytocompatibility of the scaffolds. The cells were expanded at 37 °C and 5 % CO_2_ in high-glucose Dulbecco's modified Eagle's medium (DMEM, Solarbio, China) supplemented with 10 % fetal bovine serum (FBS, Gibco, China). The scaffold was immersed in 75 % ethanol for 1 h, and then irradiated under an ultraviolet lamp for 1 h to achieve thorough sterilization. The cells were then seeded on the scaffolds and cultured *via* a 3D culture device (CellDancer 02, Ubbiotech, China). First, the scaffolds were placed into a 50 mL bioreactor tube with a 0.22 μm vent cap (NEST, China). Then, 10 ml of cells at a density of 5 × 10^4^ cells/ml were planted in the bioreactor tube containing the scaffold. The 3D culture device was set to tilt repeatedly by 45° for 2 min every 7 min for 12 h. After that, the scaffolds with cells were transferred to 24-well plates for continued culture. After 3 and 5 days of culture, cell *via*bility was determined with a CCK-8 kit (Beyotime, China) and a calcein-AM kit (Beyotime, China). The morphology of the cells on day 5 was observed *via* laser confocal scanning microscopy (CLSM) after TRITC-phalloidin (Solarbio, China) and DAPI (Solarbio, China) staining.

The PGCL, PGCL@1 %Van, PGCL@3 % Van, PGCL@5 % Van, and PGCL@10 % Van scaffolds were individually immersed in 2 ml of DMEM medium for 48 h. Subsequently, the extracted solutions were used to culture MC-3T3-E1 cells in 96-well plates. CCK-8 values were measured at 3 and 5 days, and cell viability(%) was calculated using formula (2).

(2) $$The\;cell\;viability\;\left(\%\right)=\frac{DS\left(OD450\right)}{DP\left(OD450\right)}✕\;100\%$$

where *DS* is the OD_450_ of samples containing Van and *DP* is the OD_450_ of PGCL group.

IF experiments for Runx2 and COL-I were performed. Alexa 546 labelled phalloidin (Beyotime, China) was used to stain the cytoskeleton, and DAPI was used to stain the cell nucleus. Images were captured *via* a fluorescence upright microscope (Zeiss A1, Germany), and mean fluorescence intensity was quantified using ImageJ software (NIH, US).

### 2.9 Osteogenic differentiation

The relative alkaline phosphatase (ALP) level was used to analyse the osteogenic differentiation of cells grown on the scaffolds. The 3D-cultured scaffolds with MC-3T3-E1 cells were transferred to 24-well plates and cultured for another 7 days. After being washed once with PBS, the cells on the scaffolds were fully lysed with lysis buffer (Solarbio, China) and subjected to freeze‒thaw cycles. After centrifugation at 13,000 rpm for 10 min at 4 °C, the supernatant was collected for the detection of ALP and BCA. The ALP content was determined *via* an ALP Assay Kit (Beyotime, China). The total protein content was determined *via* a BCA Assay Kit (Beyotime, China). The ALP level was calculated *via* formula (3):

(3) $$ALP\;level=ALP_{OD405}/TP_{OD562}$$

where *ALP*_*OD405*_ is the detection value of ALP and *TP*_*OD562*_ is the detection value of BCA.

### 2.10 Mineralization of osteoblasts

Calcium quantification after alizarin red S (ARS) staining was used to evaluate the osteoblast mineralization of the scaffolds. The scaffolds mentioned in the osteogenic differentiation section were cultured for 14 days in 24-well plates. After being rinsed three times in PBS, the cells on the scaffolds were fixed with 4 % paraformaldehyde (PFA) for 20 min. The cells were then stained with ARS staining solution (Beyotime, China) for 2 min, followed by thorough washing with PBS. Cetylpyridine chloride (CPC) at 10 % (w/v) was used to dissolve the ARS chelated on the calcium nodules. The absorbance of the solution at 540 nm was detected to determine the degree of osteoblast mineralization.

### 2.11 Animal experiments

Thirty-two male SD rats (weighing 150–180 g) were randomly divided into four groups (PGCL, PGCL@5 %Van, PGCL@5 %Ti_3_C_2_, and PGCL@5 %Ti_3_C_2_/5 %Van) for acute infected bone defect regeneration experiments, with 5 rats in each group. All the animal studies were conducted in accordance with the principles and procedures outlined in ‘‘Regulations for the Administration of Affairs Concerning Laboratory Animals”, approved by the National Council of China on October 31, 1988, and ‘‘The National Regulation of China for Care and Use of Laboratory Animals”, promulgated by the National Science and Technology Commission of China, on November 14, 1988, as Decree No. 2. Protocols were approved by the Committee of Jilin University Institutional Animal Care and Use. First, after being weighed, the animals were anaesthetized *via* an intraperitoneal injection of 3.5 % (w/v) sodium pentobarbital (1 ml/kg). After anaesthesia, the hair on the medial side of the proximal tibia of each animal was removed. The skin and blood vessels were peeled off with a scalpel. The medial proximal tibia on both sides was separated and exposed. A penetrating bone defect was created *via* a 3 mm diameter drill below the tibial insertion of the medial ligament. Then, 10 μl of bacterial mixture (10^8^ CFU/L) was injected into the bilateral defects. Different groups of scaffolds were used to fill the bone defect. Surgical suturing of the skin tissue outside the tibia was performed. Two animals from each group were sacrificed after 4 and 8 w, respectively. The bilateral tibias were removed and immersed in 4 % PFA.

To demonstrate the anti-infective ability of the scaffolds, 0.5 mL of blood was collected from the tail vein 3 and 7 days after surgery. The number of leukocytes in the serum of the animals after scaffold implantation was analysed with an automatic blood cell analyser (Dimay DF62 lab, China). Furthermore, IF staining for TNF-α and IL-6 was performed in samples after 1 week and 4 weeks. Bacteria within the tissues were visualized *via* Gram staining at 1 week and 3 weeks.

Micro-CT scanning was employed to analyse the repair effect on the bone defect. Then, the bone samples were decalcified and stained with hematoxylin‒eosin (H&E) and Masson's trichrome. The bone volume (BV)/tissue volume (TV) of the defect location was calculated *via* CTAn software (Bruker). H&E staining of the heart, liver, spleen, lung and kidney of rats implanted with different groups of scaffolds after 8 w was performed to observe toxicity *in vivo*. The group without scaffold implantation was referred to as the Sham group, and the group implanted with PGCL@5 %Ti_3_C_2_/5 %Van was set as the experimental group. At 8 weeks postimplantation, blood chemistry and immune marker analyses were performed on rats to investigate whether the deposition of MXene particles in the scaffold would cause organ toxicity and immune responses.

### 2.12 Transcriptomic profile of the PGCL@5 %Ti_3_C_2_ scaffolds

MC-3T3-E1 cells were cultured on PGCL and the PGCL@5 %Ti_3_C_2_ scaffold at a density of 5 × 10^4^ cells/mL for 14 days as described above. Total RNA was then extracted from the cells with Tri Quick Reagent (Solarbio, China). The concentration and purity of total RNA were assessed by a nanoplate reader (M200, Tecan). The samples were then sent to BGITech Co., Ltd., to establish two groups of libraries (PGCL and PGCL@5 %Ti_3_C_2_). These libraries were generated and sequenced *via* the Illumina HiSeqTM 2500 platform (BIOMARKER, China). Differentially expressed RNA analysis was performed *via* Mr. Tom software (BGITech, China). The expression heatmap, nonsupervised orthologous group (eggnog) enrichment bubble chart, and Kyoto Encyclopedia of Genes and Genomes (KEGG) pathway classification were included in this analysis.

### 2.13 Statistical analysis

The data are presented as the means ± standard de*via*tions (SDs). Student's *t*-test or one-way ANOVA was applied in accordance with the conditions used to determine significant differences after normalization. This experiment regarded a 2-tailed P value < 0.05 as statistically significant (n = 3). Origin 2019 Pro was used to process all the data.

## 3 Results and discussion

### 3.1 Characterization and cytocompatibility of PGCL@Ti_3_C_2_ scaffolds

**Fig. 1:**
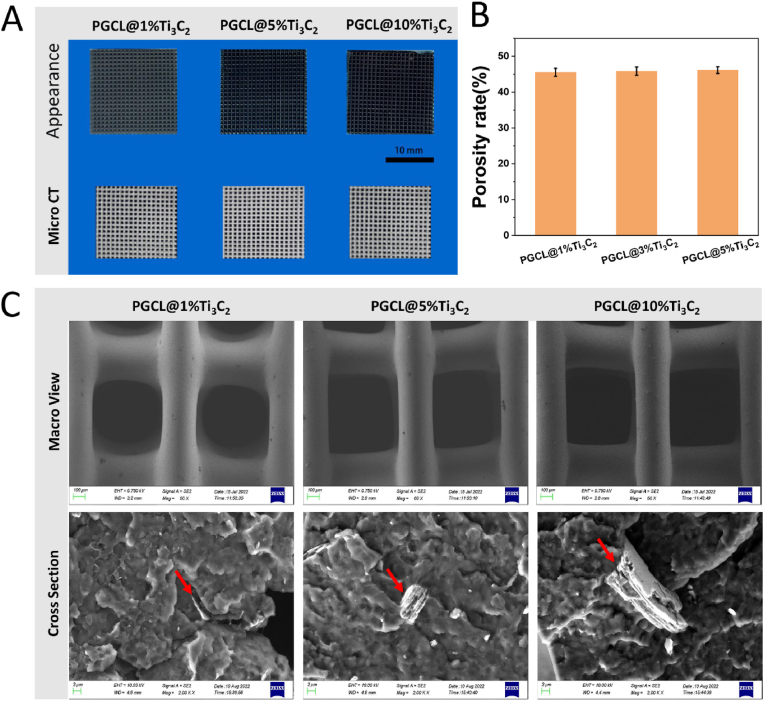
(A) Appearance and micro-CT images of the scaffolds, (B) column chart of the porosity of the scaffolds (n = 3) and (C) SEM images of the macro view and cross-section of the scaffold (the red arrow indicates Ti_3_C_2_). (For interpretation of the references to colour in this figure legend, the reader is referred to the Web version of this article.)

As shown in Fig. 1A, the macroscopic scaffolds were arranged in a regular pore structure. The colour of the scaffolds gradually increased with increasing Ti_3_C_2_ content. The 3D reconstruction model of the micro-CT image revealed that the porosity of the scaffold was determined mainly by the size and number of printed pores. The average porosity of each scaffold was calculated *via* CTAn software (Bruker). The porosities of PGCL/1 %Ti_3_C_2_, PGCL/5 %Ti_3_C_2_, and PGCL/10 %Ti_3_C_2_ were 49.3 ± 1.56 %, 48.1 ± 1.05 %, and 47.3 ± 1.83 %, respectively (Fig. 1B). There was no statistically significant difference in porosity among the groups. SEM images of the micromorphology of the scaffold surface and cross section are shown in Fig. 1C. The pore size of the scaffolds was approximately 600 μm. The scaffolds were well bonded between the layers without fracture. In the cross section of the scaffold, lamellar Ti_3_C_2_ particles were observed (indicated by red arrows). As shown in Fig. S1A, the SEM images reveal that the Ti_3_C_2_ particles exhibit a layered structure. The particle size distribution of the Ti_3_C_2_ particles ranged from 5 to 20 μm(Fig. S1B).

**Fig. 2:**
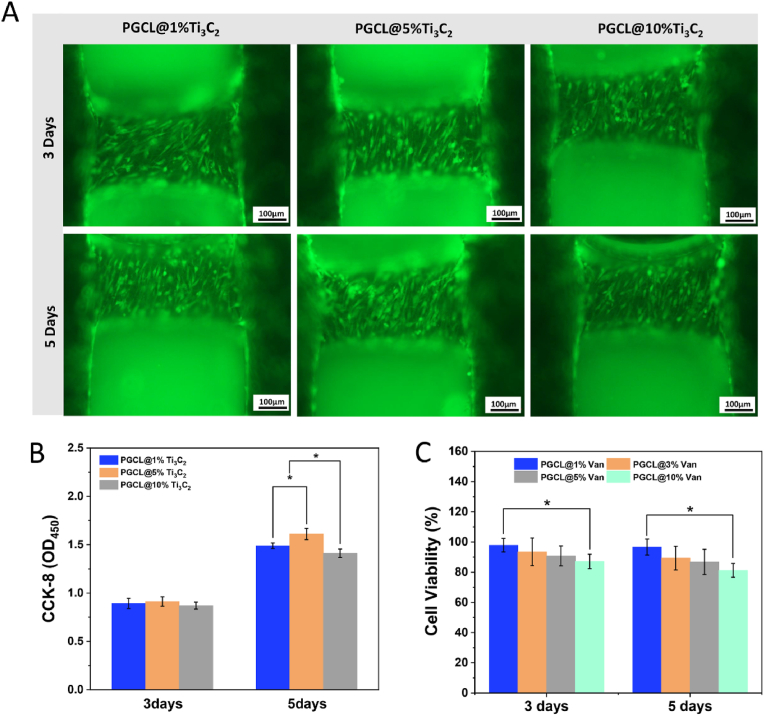
(A) Calcein AM MC-3T3-E1 cell adhesion staining, (B) CCK-8 cell proliferation assay and (C) cell viability of extracts from PGCL scaffolds with different Van contents (n = 3, ∗*P<0.05*).

As shown in Fig. 2A and B, after 3 and 5 days of growth, MC-3T3-E1 cells cultured on the scaffold surfaces were stained with calcium AM, and the cell proliferation rate was determined *via* the CCK-8 method. In all the groups, the cells attached well to the scaffold surface after 3 and 5 days. However, the scaffold containing 5 % Ti_3_C_2_ had the most cell adhesion at 5 d, while the cells on the PGCL/10 % Ti_3_C_2_ scaffold shrank, resulting in exposure of the scaffold surface. The cell proliferation results were consistent with the staining results. At 3 days, there was no significant difference in cell proliferation among the three groups. The cells in each group grew over time. On day 5, the cell proliferation of the PGCL/10 %Ti_3_C_2_ group was significantly lower than that of the PGCL/1 %Ti_3_C_2_ group and the PGCL/5 %Ti_3_C_2_ group (*P<0.05*). The cell proliferation of the PGCL/5 %Ti_3_C_2_ group was significantly greater than that of the PGCL/1 %Ti_3_C_2_ group (*P<0.05*). To further investigate the effect of Van on MC-3T3-E1 cell viability, we tested the viability of the extracts of the PGCL scaffolds containing different concentrations (1 %–10 %) of Van. As shown in Fig. 2C, on day 5, the cell viability of the 10 % Van group was 81.3 ± 4.61 %, whereas that of the 1 %–5 % Van groups was greater than 85 %.

The results of cell adhesion and proliferation showed that although PGCL had good biocompatibility, the biocompatibility of the material was still affected by the addition of MXene. Ke Chen et al. demonstrated that PLA membranes containing 0.5 % and 1 % MXene (Ti_3_C_2_Tz) nanosheets were able to promote the adhesion and proliferation of MC-3T3-E1 cells [28]. Di Cui et al. evaluated the cytocompatibility of low-content MXene (Ti_3_C_2_Tx) using periodontal ligament cells (PDLCs) and reported that 180 mg/L and 360 mg/L MXene significantly promoted cell proliferation [29]. Xu et al. reported that the addition of MXene (Ti_2_AlN) to PCL effectively improved the hydrophilicity and surface roughness of the scaffold, which promoted cell adhesion. However, the high Ti_2_AlN content (over 5 %) was disadvantageous for enhancing cell adhesion and proliferation [30]. These studies indicated that an appropriate amount of MXene in a polymer material could promote cell viability. However, excessive addition may cause cytotoxic effects. The molecular mechanism by which MXene materials promote cell adhesion and proliferation is still not clear, and further study is needed. According to this research, the adhesion and proliferation of MC-3T3-E1 cells were most effectively enhanced on the PGCL/5 %Ti_3_C_2_ scaffolds. The PGCL/10 %Ti_3_C_2_ scaffold was more cytotoxic than the PGCL/1 %Ti_3_C_2_ and PGCL/5 %Ti_3_C_2_ scaffolds. These results indicated that the PGCL/5 %Ti_3_C_2_ scaffold was more suitable for cell growth. Therefore, the content of Ti_3_C_2_ in subsequent experiments was set to 5 %.

### 3.2 Characterization and antibacterial properties of the PGCL@Van scaffolds

**Fig. 3:**
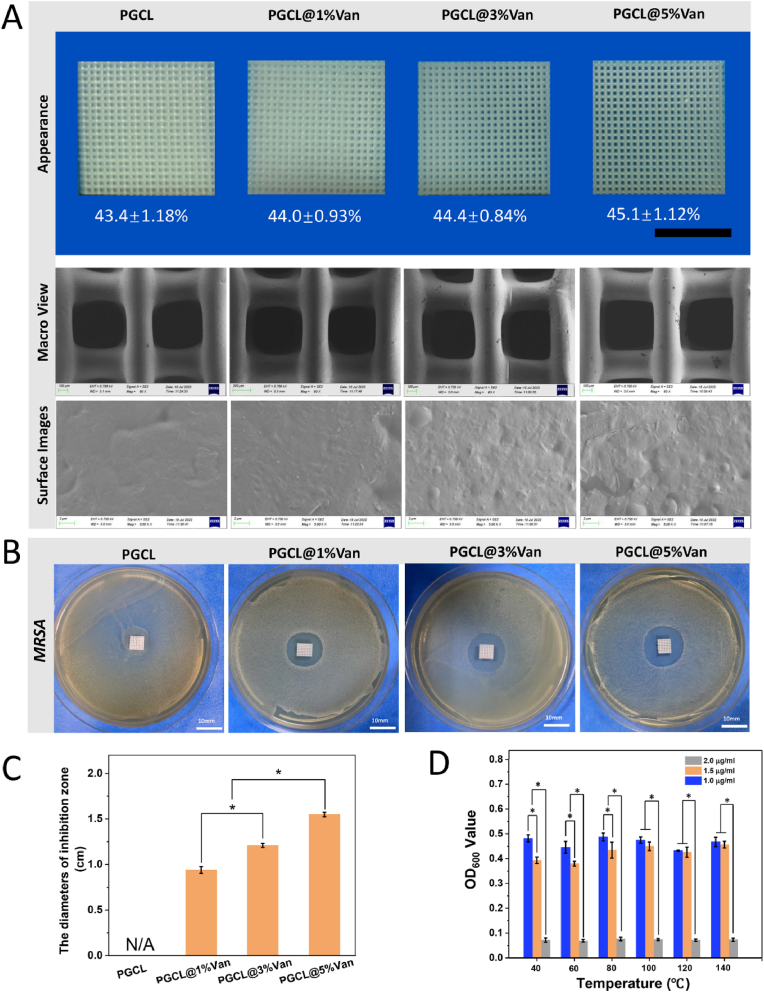
(A) Appearance of the scaffold, SEM macroimages and surface images. (B and C) Inhibition zone images and histograms of the scaffold. (D) The antibacterial ability of Van after treatment at 40–140 °C, n = 3 ∗*P<0.05*.

Scaffolds containing different amounts of Van (PGCL, PGCL@1 %Van, PGCL@3 %Van, and PGCL@5 %Van) were fabricated. The Van-containing scaffolds had the same macroscopic structure and porosity as the PGCL scaffolds. With increasing Van content, the microscopic surface of the scaffolds gradually became rougher (Fig. 3A). To verify whether Van retains its antibacterial activity in the scaffolds after high-temperature printing, we studied the antibacterial ability of Van after high - temperature treatment at 40–140 °C. As shown in Fig. 3D. The antibacterial ability of 2.0 μg/mL Van was significantly greater than that of 1.0 μg/mL and 1.5 μg/mL Van after all the temperature treatments (*P<0.05*). A temperature below 140 °C had no significant effect on the antibacterial effect of Van. The antibacterial activity of the scaffolds with different Van contents was subsequently evaluated *via* the inhibition zone method. The appearances and diameters of the zones of inhibition are shown in Fig. 3B and C. The PGCL scaffold was not antibacterial. With increasing Van content, the surrounding scaffolds also gradually expanded. The diameters of the inhibition zones of the three groups (PGCL@1 %Van, PGCL@3 %Van, and PGCL@5 %Van) were 9.4 ± 0.38 mm, 12.1 ± 0.21 mm, and 15.5 ± 0.25 mm, respectively. The diameter of the inhibition zone in the PGCL@5 %Van group was significantly greater than that in the other two groups (*P<0.05*). The inhibition zone was also significantly greater in the PGCL@3 %Van group than in the PGCL@1 %Van group (*P<0.05*).

Many pathogens are associated with bone infection, among which *MRSA* is the most common and serious. Antimicrobial resistance is a major challenge in the treatment of osteomyelitis. Up to 50 % of *MRSA* osteomyelitis cases are caused by *MRSA* [31]. Van is an important drug for the clinical treatment of *MRSA* osteomyelitis because it significantly inhibits *MRSA*. In addition, it could maintain its antibacterial ability at 140 °C, which is higher than the printing temperature of the PGCL scaffold (140 °C). Therefore, Van was used as an antibacterial component to prepare the dual-functional scaffold in this study. PMMA-based antibiotic cement is a commonly used clinical bone filling material. We compared the antibacterial effects of PMMA cement and PGCL scaffolds containing different proportions of Van against *MRSA*. As shown in Fig. S2 A and B, when the Van content was 1 %–5 %, there was no significant difference in the diameter of inhibition zone between the PMMA cement and PGCL scaffolds. Both showed good effects against *MRSA* and exhibited a dose-dependent relationship. PMMA cement is a nondegradable material, so only the Van on the surface layer is released into the surrounding environment. As time increases, the concentration of Van in the bone infection area gradually decreases. In contrast, PGCL material is a biodegradable material. As PGCL degrades, Van in the scaffold is be continuously released, and this sustained release of Van produces a long-term antibacterial effect on bacteria at the site of bone infection.

### 3.3 Characterization of dual-function composite scaffolds

#### 3.3.1 Morphology

**Fig. 4:**
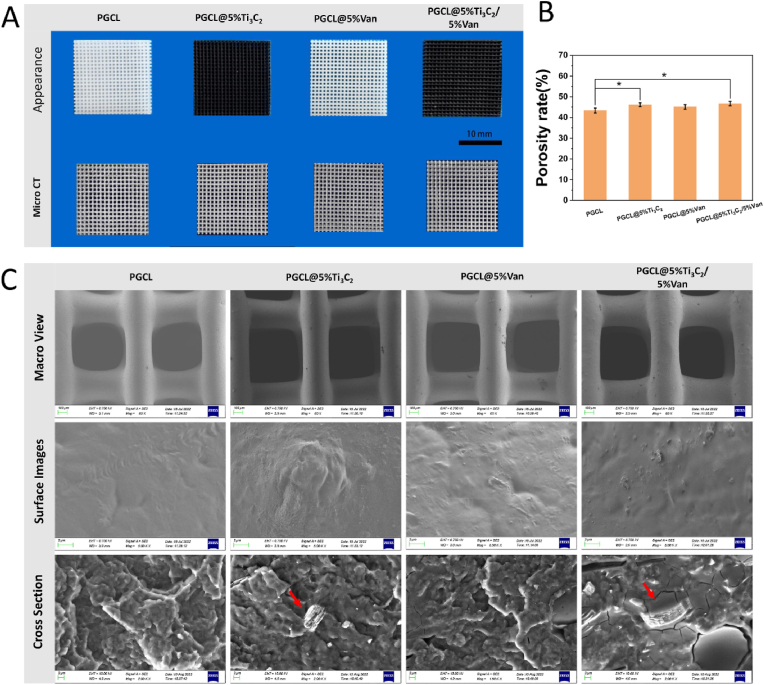
(A) Appearance of the scaffolds in the different groups and Micro CT 3D reconstruction images. (B) Porosity rate of the scaffolds in the different groups (n = 3). (C) SEM macroimages, surface and cross section images of the scaffolds in the different groups.

On the basis of the above results, the contents of Ti_3_C_2_ and Van in the dual-function scaffold were determined. Four groups (PGCL, PGCL@5 %Ti_3_C_2_, PGCL@5 %Van, and PGCL@5 %Ti_3_C_2_/5 %Van) were therefore set up for subsequent experiments. As shown in Fig. 4A, the PGCL@5 %Ti_3_C_2_/5 %Van scaffold exhibited a regular pore structure, which was comparable to that of the other scaffolds. The PGCL and PGCL@5 %Van scaffolds presented the inherent white colour of the material, whereas the scaffolds containing Ti_3_C_2_ were dark. The micro-CT results of the scaffold revealed that the internal pores of the scaffold were connected. The contrast in the porosity rates of the scaffolds is shown in Fig. 4B. The porosities of the PGCL@5 %Ti_3_C_2_ and PGCL@5 %Ti_3_C_2_/5 %Van scaffolds were 46.0 ± 2.28 % and 48.1 ± 1.05 %, respectively. The porosities of these scaffolds containing Van and Ti_3_C_2_ were higher than that of PGCL (44.1 ± 2.01 %) (*P<0.05*). The porosity of the PGCL@5 %Van scaffold was 44.8 ± 1.68 %, which was not significantly different from that of the other groups. The addition of Van and Ti_3_C_2_ to the PGCL had a certain effect on the viscosity of the PGCL. This phenomenon increases the shear force during extrusion of the material, resulting in thinning of the fibers and increase in scaffold porosity. SEM images of the micromorphology of the scaffold surface and cross section are shown in Fig. 4C. The surface of the PGCL was smooth, and the cross section also presented a homogeneous state. In the groups containing Ti_3_C_2_, the Ti_3_C_2_ particles (indicated by red arrows) could be observed both on the surface and in the cross section. The surface of PGCL@5 %Van was also rougher than that of PGCL. In addition, small cracks were observed on the cross section of the scaffolds containing Van. The pore size of all the scaffolds was approximately 600 μm. The scaffolds were well bonded between the layers without fracture.

#### 3.3.2 Structure and viscosity

**Fig. 5:**
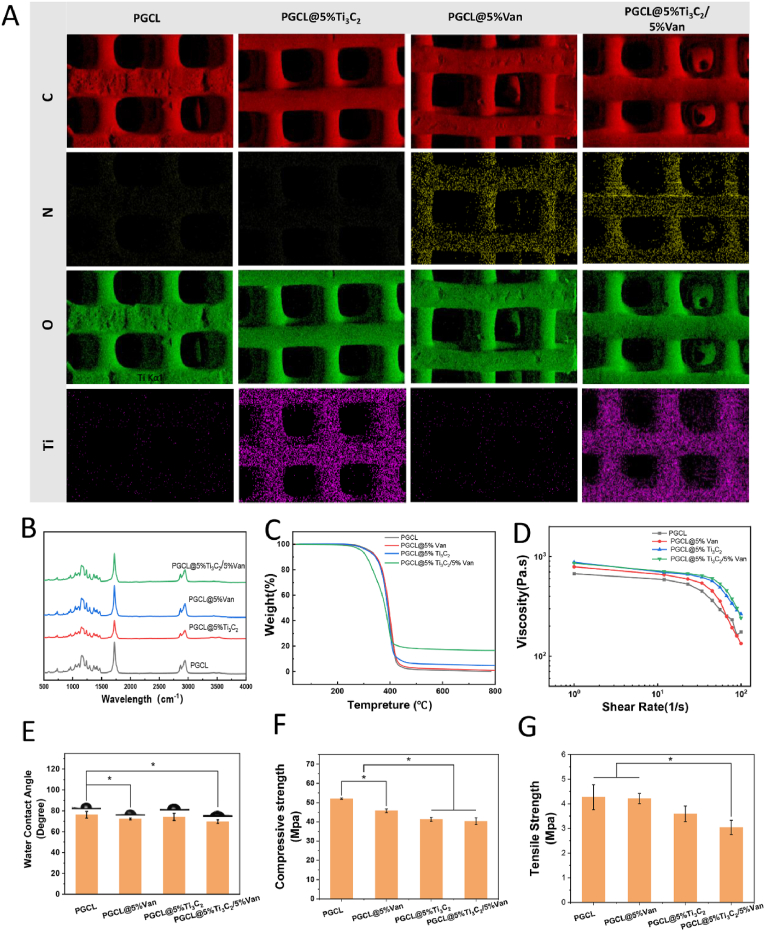
(A) EDS mapping, (B) FTIR and (C) TGA analysis of the chemical composition of the scaffold surface. (D) Viscosity and (E) WCA of the different groups. (F) Compressive strength and (G) tensile strength of the scaffold, n = 3 ∗*P* < 0.05.

The composite scaffold is composed of PGCL, Van, and MXene, which may contain C, N, O, and Ti. As shown in Fig. 5A, EDS mapping was used to scan and evaluate the element distributions of C, N, O and Ti on the scaffold surface. Although C and O are contained in Van and Ti_3_C_2_, they are derived mainly from the PGCL substrate. There was no significant difference in the C or O contents among the four groups. Van contains the characteristic element of N. Ti_3_C_2_ contains the characteristic element of Ti. Both elements were relatively evident and evenly distributed in their respective scaffolds.

As shown in Fig. 5B, FTIR characterization of the composites was performed. The stretching vibration of the –CH_2_- skeletal group appeared in the 3000-2800 cm^−1^ and 1500-1300 cm^−1^ regions. The stretching vibration of C=O bonds appeared in the region near 1720 cm^−1^. The stretching vibrations of the C-O-C and C-O groups appeared in the 1050-1250 cm^−1^ region. These bonds are characteristic of aliphatic polyesters and are the same in each group. The intensity of the characteristic peaks in the Ti_3_C_2_-containing group decreased, whereas that of PGCL@5 %Van did not significantly differ from that of PGCL.

As shown in Fig. 5C, the weight loss rates of PGCL, PGCL@5 %Ti_3_C_2_, PGCL@5 %Van, and PGCL@5 %Ti_3_C_2_/5 %Van at a peak of 431 °C were 94.715 %, 93.023 %, 90.952 % and 79.913 %, respectively. At a peak of 431 °C, most of the PGCL and van in the material evaporated, and the remaining component was Ti_3_C_2_. The TGA results revealed that Ti_3_C_2_ was successfully incorporated into PGCL.

As shown in Fig. 5D, the viscosity of the raw materials was measured *via* an MCR rheometer. Within the shear rate range of 0–12 1/s, the materials exhibited Newtonian plateau behaviour. When the shear rate increased to 12–100 1/s, the viscosity of each material decreased, demonstrating a shear-thinning phenomenon. At a shear rate of 12 1/s, the viscosities of PGCL@5 %Ti_3_C_2_, PGCL@5 %Van, and PGCL@5 %Ti_3_C_2_/5 %Van were 1.12, 1.18, and 1.21 times greater than that of PCL, respectively. The viscosity of the composite scaffold containing Ti_3_C_2_ was greater than that of the PGCL. The viscosity of the material was beneficial for maintaining the sample shape and avoiding the collapse of the beam frame during printing. The increase in the viscosity of the material containing Ti_3_C_2_ affects the printing process, which manifests mainly as the thinning of the scaffold fibers and an increase in porosity (Fig. 4B).

#### 3.3.3 Hydrophilicity

As shown in Fig. 5E, the contact angles of PGCL, PGCL@5 %Van, PGCL@5 % Ti_3_C_2_, and PGCL@5 %Ti_3_C_2_/5 %Van were 76.2 ± 3.21°, 72.2 ± 0.79°, 74.2 ± 3.39° and 69.7 ± 1.71°, respectively. The contact angles of the scaffolds containing Van (PGCL@5 %Van or PGCL@5 %Ti_3_C_2_/5 %Van) were significantly lower than those of the PGCL. This indicated that Van improved the hydrophilicity of the PGCL. Malika Ardhaoui et al. systematically studied the relationship between the contact angle and osteoblast adhesion, and the experimental results revealed that the material surface with a contact angle of 65° was most suitable for the adhesion and growth of osteoblasts [32]. Although the surface of the PGCL scaffold exhibited reasonable hydrophilicity for cell growth, the contact angle of PGCL@5 %Ti_3_C_2_/5 %Van was close to 65°, which was more conducive to cell adhesion.

#### 3.3.4 Mechanical properties

A comparison of the compression strength and tensile strength between the groups is shown in Fig. 5F and E. The compressive strengths of PGCL, PGCL@5 %Van, PGCL@5 %Ti_3_C_2_, and PGCL@5 %Ti_3_C_2_/5 %Van were 52.0 ± 0.37 MPa, 45.7 ± 0.99 MPa, 41.3 ± 0.97 MPa, and 40.3 ± 1.75 MPa, respectively. The difference between the PGCL group and the other groups was significant (*P<0.05*). In addition, the compressive strength of the PGCL@5 %Van scaffold was greater than that of the PGCL@5 %Ti_3_C_2_ and PGCL@5 %TI_3_C_2_/5 %Van scaffolds, and the difference was statistically significant (*P<0.05*). The results indicated that the compressive strength of the scaffolds decreased with the addition of van and Ti_3_C_2_ and that, compared with van, Ti_3_C_2_ had a greater effect on the compressive strength. The tensile strengths of PGCL, PGCL@5 %Van, PGCL@5 %Ti_3_C_2_, and PGCL@5 %TI_3_C_2_/5 %Van were 4.3 ± 0.51 MPa, 4.2 ± 0.20 MPa, 3.6 ± 0.31 MPa, and 3.0 ± 0.29 MPa, respectively. The tensile strength of the scaffolds containing Ti_3_C_2_ was lower than that of those containing PGCL or PGCL@5 %Van. However, only PGCL@5 %Ti_3_C_2_/5 %Van was significantly different between these two groups (*P<0.05*). The results indicated that the tensile strength of the scaffolds decreased with the addition of Ti_3_C_2_.

The appropriate mechanical strength of materials is conducive to bone repair. The present results showed that the tensile strength and compressive strength significantly decreased when Ti_3_C_2_ particles were added to PGCL. Some previous studies have shown that mixing a certain proportion of MXene into polymer materials could enhance the mechanical strength of composite materials [33]. However, when a certain amount of MXene was added, the mechanical strength of the material decreased. Ke Chen et al. prepared OTES-Ti_3_C_2_Tz/PLA nanocomposite membranes containing different contents of OTES-Ti_3_C_2_Tz. SEM images revealed that OTES-Ti_3_C_2_Tz aggregated when the content of OTES-Ti_3_C_2_Tz exceeded 0.5 %, and the tensile strength also decreased [28]. Previous studies have demonstrated that the tensile strength of cancellous bone in the femur ranges from 0.986 to 2.648 MPa, whereas its compressive strength ranges from 2.961 to 4.018 MPa. In this study, the PGCL@5 %Van/5 %Ti_2_C_3_ composite scaffold exhibited tensile and compressive strengths of 3.0 ± 0.29 MPa and 40.3 ± 1.75 MPa, respectively. These values either meet or exceed the mechanical properties of natural cancellous bone, indicating its potential applicability for bone repair in cancellous bone regions of the femur or other nonload-bearing skeletal areas [34].

### 3.4 Antibacterial activity and drug release of the dual-function composite scaffold

**Fig. 6:**
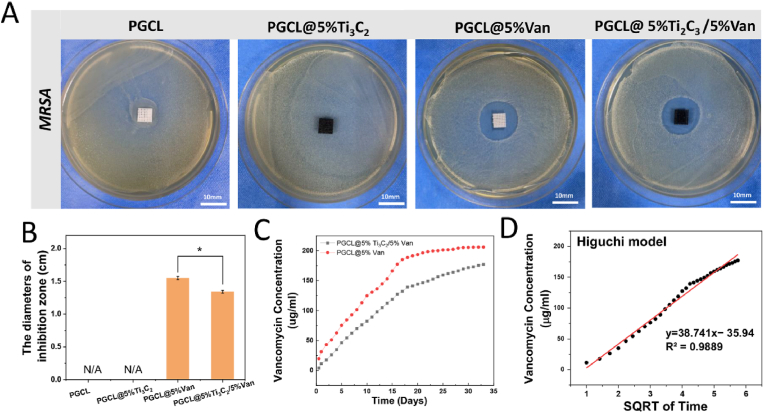
The images of (A) the inhibition zone and (B)the bar chart of the diameters of the inhibition zone for the scaffolds from different groups. (C) The release curve of Van from the scaffold and (D) the Higuchi model of Van release from the PGCL@5 %Ti_3_C_2_/5 %Van scaffold, n = 3 ∗*P* < 0.05.

The antibacterial activity of the scaffolds was evaluated *via* the inhibition zone method. The appearances and diameters of the zones of inhibition are shown in Fig. 6A and B. The PGCL and PGCL@5 %Ti_3_C_2_ scaffolds were not antibacterial. The diameters of the inhibition zones of PGCL@5 %Van and PGCL@5 %Ti_3_C_2_/5 %Van were 15.5 ± 0.25 mm and 13.4 ± 0.22 mm, respectively. The diameter of the inhibition zone in the PGCL@5 %Van group was significantly greater than that in the PGCL@5 %Ti_3_C_2_/5 %Van group (*P<0.05*). The Van release curves of the PGCL@5 %Van and PGCL@5 %Ti_3_C_2_/5 %Van scaffolds are shown in Fig. 6C. The values in the release curves were calculated *via* the standard curve equation shown in Fig. S3. In the PGCL@5 %Ti_3_C_2_/5 %Van group and the PGCL@5 %Van group, Van showed a sustained release effect from the scaffolds throughout the 33-day release period. Between day 18 and day 33, the amount of Van released decreased slightly. The drug release in the PGCL@5 %Van group was consistently greater than that in the PGCL@5 %Ti_3_C_2_/5 %Van group at all time points. The Ti_3_C_2_ particles in the scaffold delayed the release rate of Van. However, according to the calculations, the drug concentration released by the scaffolds in the two groups was always greater than 2 μg/mL. As shown in Fig. 6D, the Higuchi model (R^2^ value = 0.9889) was the best fit for the release process of Van from the PGCL@5 %Ti_3_C_2_/5 %Van scaffold. The model assumes that during the entire 33-day release period, Van is released from the pores of the PGCL@5 % Ti_3_C_2_/5 %Van scaffold, where Van diffuses and is released in a time-dependent manner.

*In situ* bone repair scaffolds containing antibiotics can be regarded as targeted delivery carriers to retain drug activity and prolong drug release. The sustained release of antibiotics can continuously suppress infection in bone defects. However, the local burst release of antibiotics may cause cytotoxicity [35]. In the present study, the PGCL@5 %Ti_3_C_2_/5 %Van scaffold promoted the controlled release of Van. Although the PGCL@5 %Van scaffold was more effective than the PGCL@5 %Ti_3_C_2_/5 %Van scaffold was, both showed outstanding antibacterial activity against *MRSA*. The MXene material is considered an effective antibacterial material [19,36]. Ahmad Arabi Shamsabadi et al. investigated the antimicrobial ability of Ti_3_C_2_Tx MXene nanosheets against *B. subtilis* and *E. coli*. The results indicated that direct physical interactions between the sharp edges of the nanosheets and bacterial membrane surfaces play a significant role in the antibacterial properties of the nanosheets. The antibacterial activity of MXenes is size dependent. Notably, the treatment of *B. subtilis* and *E. coli* with 0.09–4.40 μm Ti_3_C_2_Tx MXene nanosheets for 8 h resulted in an antibacterial rate greater than 90 % [37]. According to our study, Ti_3_C_2_ did not exhibit antibacterial properties. This may be due to the large particle size of the material we used (mainly in the range of 8–15 μm). In addition, the nanosheet structure of Ti_3_C_2_ contributed to the sustained release of Van.

### 3.5 Degradation and biomimetic mineralization *in vitro*

**Fig. 7:**
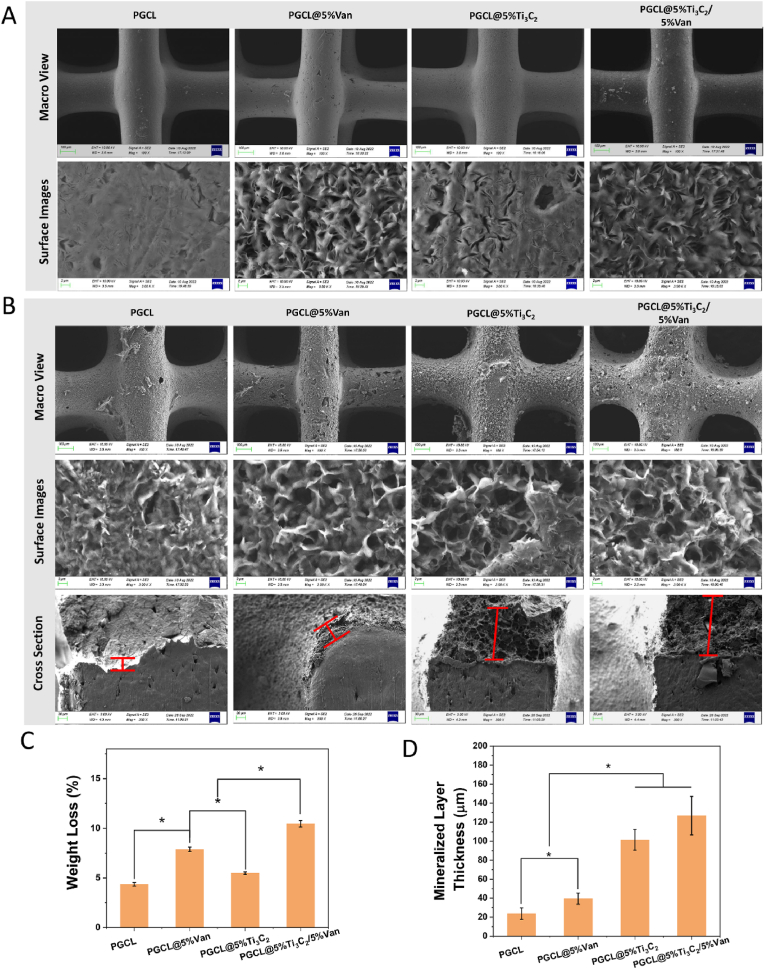
(A) SEM images of the scaffold after degradation. (B) SEM images of the scaffold after *in vitro* mineralization (the red line indicates the mineralization layer.). (C) Weight loss of the scaffold after degradation and (D) thickness of the mineralization layer on the scaffold, n = 3 ∗*P* < 0.05. (For interpretation of the references to colour in this figure legend, the reader is referred to the Web version of this article.)

The surface morphology and weight loss of different scaffolds after the degradation of lipase were investigated. As shown in Fig. 7A, after the scaffolds were degraded in the presence of lipase for 15 days, cracks appeared on the surfaces of the scaffolds in the PGCL and PGCL@5 %Ti_3_C_2_ groups. However, many microporous structures emerged on the surfaces of the scaffolds in the PGCL@5 %Van and PGCL@5 %Van/5 %Ti_3_C_2_ groups which contained Van.

The weight loss of the different scaffolds is shown in Fig. 7C. The weight loss of each group gradually increased significantly (PGCL@5 %Van/5 %Ti_3_C_2_> PGCL@5 %Van > PGCL@5 %Ti_3_C_2_>PGCL, *P<0.05*). The accelerated degradation rate of the scaffolds containing Van may be related to the increased hydrophilicity of the scaffolds. The contact angle results of the scaffolds revealed that the hydrophilicity of the scaffolds containing Van was significantly greater than that of the PGCL group (Fig. 5E). The enhanced hydrophilicity of the scaffold surface accelerated the process of the lipase-mediated hydrolysis reaction [38]. Similar to most biodegradable polymers, the degradation process of PGCL *in vivo* can be divided into two stages. First, PGCL was enzymatically hydrolysed to short-chain polymers. After that, the short-chain polymers continue to degrade and eventually become small molecules that can be digested by cells or excreted outside [16,39].

SEM images of the morphology of biomimetic mineralization on the scaffolds are shown in Fig. 7B. Dense mineralized layers were observed on the surface of all the materials. There were more mineralized nodules observed on the surface of the Ti_3_C_2_-containing materials than in the other two groups. The thickness of the mineralized layers is marked by the red line segments in the cross-sectional photos, and the measurement results are shown in Fig. 7D. The thicknesses of the mineralized layers on the surfaces of PGCL, PGCL@5 %Van, PGCL@5 %Ti_3_C_2_, and PGCL@5 %TI_3_C_2_/5 %Van were 23.7 ± 5.98 μm, 39.5 ± 5.89 μm, 101.4 ± 10.88 μm, and 126.9 ± 20.22 μm, respectively. The thickness of the mineralized layers on the surface of the Ti_3_C_2_-containing materials was significantly greater than that of the other two materials (*P<0.05*). The mineralized layer on the PGCL/5 %Van scaffold was significantly thicker than that on the PGCL scaffold (*P<0.05*).

The formation ability of apatite is critical for evaluating the bone-promoting ability of materials and facilitating bone remodelling. The results of our study revealed that apatite could form on the surface of each scaffold. The addition of Ti_3_C_2_ and Van enhanced the mineralization ability of PGCL. The mechanism of enhanced mineralization ability could be attributed mainly to rough surfaces, which provide many sites of mineralization.

### 3.6 Osteogenesis activity *in vitro*

**Fig. 8:**
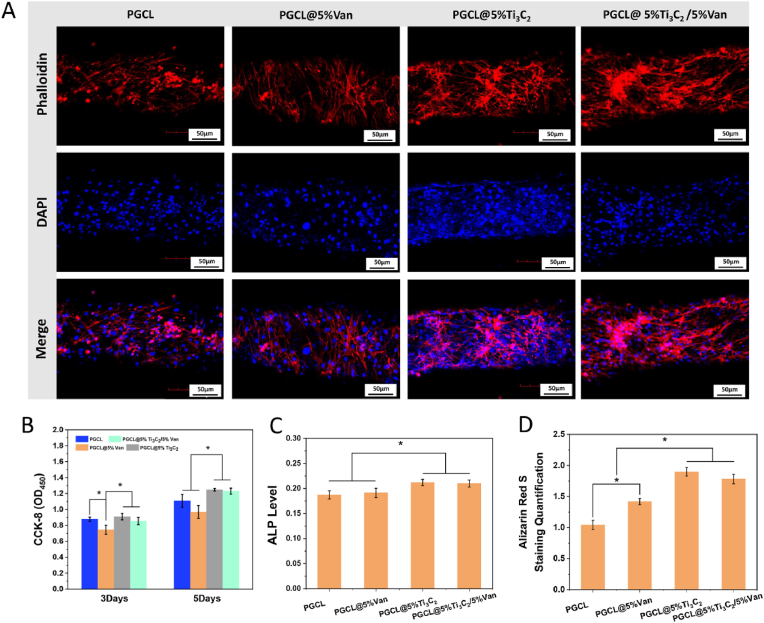
(A) Cells cultured on different scaffolds stained with phalloidin (red indicates the cytoskeleton, the blue denotes the nucleus). (B) CCK-8 assay. (C) ALP level assay. (D) ARS quantification, n = 3 ∗*P* < 0.05. (For interpretation of the references to colour in this figure legend, the reader is referred to the Web version of this article.)

The cell morphology is shown in Fig. 8A. The cytoskeleton and nucleus of MC3T3-E1 cells were dyed red and blue, respectively. The cells were evenly expanded on the scaffolds of each group with a stretched morphology. The cells in the PGCL group presented an elongated morphology, and no obvious ordered actin filaments were observed. In the PGCL@5 %Ti_3_C_2_ and PGCL@5 %Ti_3_C_2_/5 %Van groups, the cells exhibited a polygonal morphology, and ordered actin filaments could be observed in the cells. The fluorescence intensity and area of the PGCL@5 %Ti_3_C_2_ and PGCL@5 %Ti_3_C_2_/5 %Van groups were greater than those of the other two groups, whereas those of the PGCL@5 %Van group were the lowest. The results of the CCK-8 test shown in Fig. 8B revealed that the cells on the scaffolds in each group tended to proliferate over time. After 3 days of culture, there was no difference in cell proliferation among the PGCL, PGCL@5 %Ti_3_C_2_ and PGCL@5 %Ti_3_C_2_/5 %Van groups, whereas the cell proliferation of the PGCL/5 %Van group was significantly lower than that of the other groups (*P<0.05*). After 5 days of culture, cell proliferation was significantly greater in the Ti_3_C_2_-containing group than in the other two groups (*P<0.05*). The PGCL@5 %Ti_3_C_2_/5 %Van scaffold enhances the adhesion and growth of osteoblasts.

Good cytocompatibility and the ability to promote cell proliferation are the basic requirements of bone repair materials. Although PGCL is a biocompatible material, its cell adhesion ability is poor. While Van was antibacterial, it also inhibited cell growth on the material. As mentioned above, many reports have shown that MXene materials can promote cell growth [[28], [29], [30]]. According to the present study, Ti_3_C_2_ not only promoted cell *via*bility but also reversed the negative effects of Van on cell proliferation. The PGCL@5 %Ti_3_C_2_/5 %Van scaffold showed good cytocompatibility and met the basic requirements of bone repair materials.

Osteogenic differentiation on the scaffolds was evaluated through the ALP level. As shown in Fig. 8C, after 5 days of culture, the ALP levels of the PGCL@5 %Van, PGCL@5 %Ti_3_C_2_, and PGCL@5 %TI_3_C_2_/5 %Van groups were 1.05, 1.31, and 1.29 times greater than those of the PGCL group, respectively. It was significantly greater in the Ti_3_C_2_-containing groups than in the other two groups (*P<0.05*). The above results indicated that the addition of Ti_3_C_2_ to PGCL promoted osteogenic differentiation in MC3T3-E1 cells. The capacity of MXene to promote osteogenic differentiation has been demonstrated in many studies [[24], [25], [26], [27]], which was confirmed in our study. This function of composite materials will be beneficial for bone defect repair.

Quantitative analysis of ARS staining can detect the calcium level of osteoblasts in the late stage. In addition, extracellular calcium deposition is a marker of osteoblast maturation, which contributes to bone remodelling [40]. According to the results shown in Fig. 8D, after 14 days of culture, the ARS staining results of the PGCL@5 %Van, PGCL@5 %Ti_3_C_2_, and PGCL@5 %TI_3_C_2_/5 %Van groups were 1.40, 1.76, and 1.57 times greater than those of the PGCL group, respectively. This value was significantly greater in the PGCL@5 %Ti_3_C_2_ and PGCL@5 %Ti_3_C_2_/5 %Van groups than in the other two groups (*P<0.05*). ARS staining was also greater in the PGCL@5 %Van group than in the PGCL group. These results demonstrated the advantages of composite materials in promoting extracellular calcium deposition in osteoblasts. In addition to the osteogenic effect of Ti_3_C_2_, the roughness of the material surface caused by the addition of Van was also a reason for the increase in extracellular calcium deposition. The advantage of the PGCL@5 %TI_3_C_2_/5 %Van scaffold in terms of osteoblast calcium deposition is that it promotes bone maturation in the later stage of bone injury repair.

### 3.7 Anti-infection effects of the dual-function composite scaffold *in vivo*

The number of leukocytes in the serum of the rats after implantation is shown in Table S2. The number of leukocytes in all the groups increased over time, indicating that an infection occurred. There were fewer leukocytes in the PGCL@5 %Ti_3_C_2_ group than in the blank and PGCL groups on day 3. However, on day 7, the number of leukocytes in the PGCL@5 %Ti_3_C_2_ group, which was equal to that in the PGCL group, was lower than that in the blank group. In the groups containing Van, the number of leukocytes sharply decreased at 3 and 7 days after implantation.

**Fig. 9:**
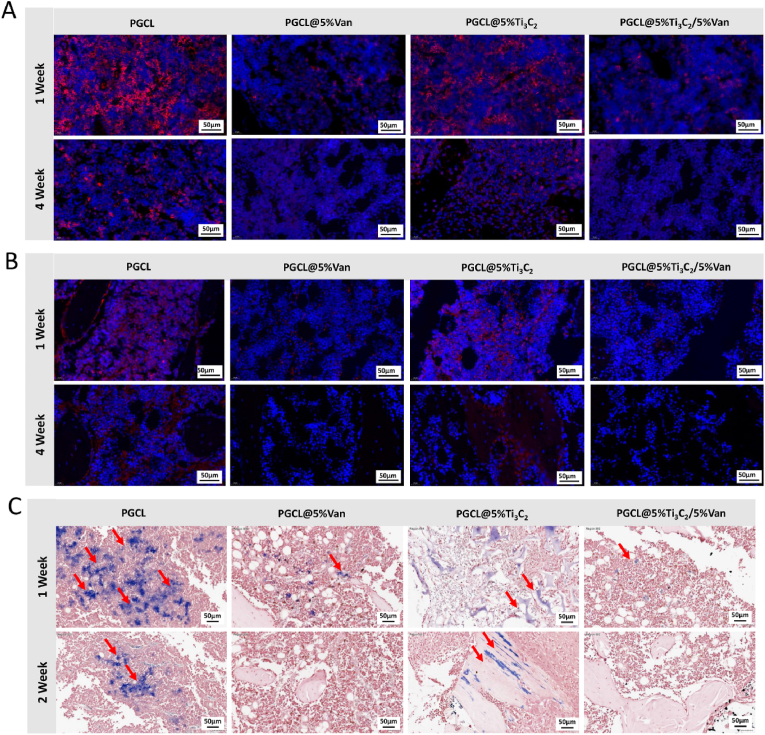
IF analysis of (A) IL-6 and (B) TNF-α. (C) Gram-stained histochemical analysis (the purple dyed regions represent bacteria). (For interpretation of the references to colour in this figure legend, the reader is referred to the Web version of this article.)

In the early stage of bone infection, the levels of IL-6 and TNF-α are increased. They are directly involved in bone destruction during bone infection and the regulation of osteoclast activity and play a role in the pathogenesis of infected bone defects [37]. As shown in Fig. 9A and B, changes in IL-6 and TNF-α in infected bone defects at 1 and 4 weeks after surgery were observed. The levels of inflammatory factors in all the groups decreased over time. Compared with those in the other three groups, the levels of IL-6 and TNF-α in the PGCL group were the highest. The levels of these two inflammatory factors in the groups treated with Van were equal and the lowest. The levels of inflammatory factors were only slightly lower in the PGCL@5 %Ti_3_C_2_ group than in the PGCL group but were significantly greater than those in the other two groups.

The results of rapid Gram staining of the tissue are shown in Fig. 9C. Purple-dyed bacteria were observed. Compared with that in the Van-containing group, the infection of the PGCL and PGCL@5 %Ti_3_C_2_ groups was significantly aggravated, and local tissue destruction was obvious. The PGCL@5 %Van and PGCL@5 %Ti_3_C_2_/5 %Van groups presented outstanding antibacterial effects, and the infection situation was significantly reversed at the 2 nd week, indicating that bacterial growth was inhibited by the sustained release of loaded antibiotics. These results strongly demonstrated that the PGCL@5 %Ti_3_C_2_/5 %Van scaffold could effectively inhibit bacterial infection, especially that caused by *MRSA*, in an acute-infected bone defect model.

### 3.8 Bone repair of dual-function composite scaffolds *in vivo*

**Fig. 10:**
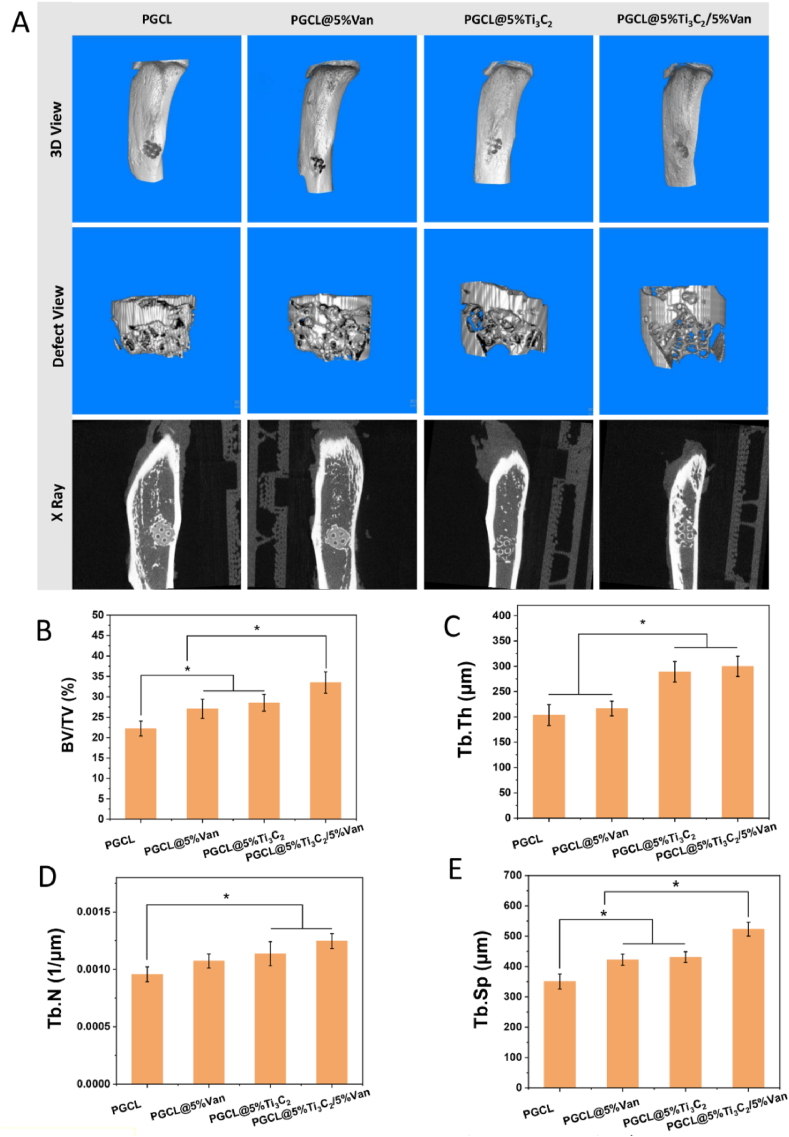
(A) Micro CT and X-ray images of rat tibial defect repair. (B–E) BV/TV, Tb.Th, Tb.N, and Tb.Sp of the newly formed bone, n = 3 ∗*P* < 0.05.

The process of establishing the rat tibial defect model and scaffold implantation is shown in Fig. S4(A–C), and the appearance of the repaired rat tibia is shown in Fig. S4(D). As shown in Fig. 10A, the micro-CT and X-ray results revealed that different scaffolds exhibited different osteogenic effects *in vivo*. X-ray images revealed that PGCL@5 %Ti_3_C_2_/5 %Van resulted in new bone formation in the scaffolds, whereas the other groups of scaffolds resulted in new bone formation mainly around the scaffolds. Macro3D and defect view images revealed that the cortical bone at the defect site was essentially repaired in all the groups. As shown in Fig. 10B–E, Micro-CT data analysis revealed that the BV/TV, trabecular thickness (Tb.Th), trabecular separation (Tb.sp) and trabecular number (Tb.N) in the PGCL@5 %Ti_3_C_2_/5 %Van group were significantly greater than those in the PGCL and PGCL@5 %Van groups (*P<0.05*). In addition, the BV/TV value and Tb.Sp in the PGCL@5 %Ti_3_C_2_/5 %Van group were also significantly greater than those in the PGCL@5 % Ti_3_C_2_ group (*P<0.05*).

**Fig. 11:**
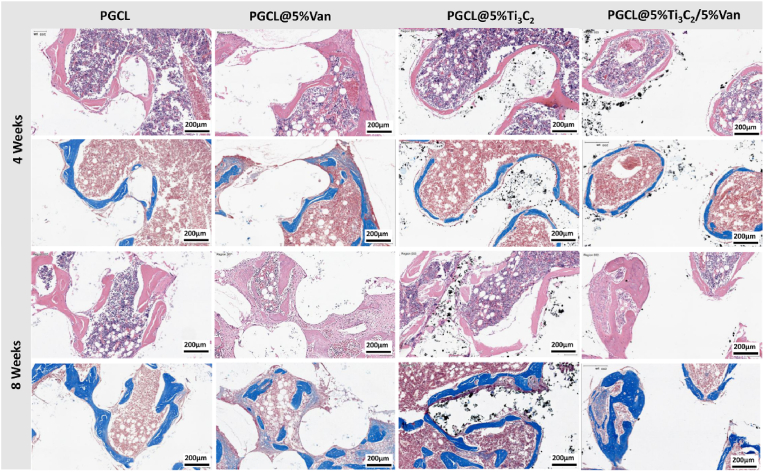
H&E and Masson staining of rat tibial defect sites implanted with different groups of scaffolds.

The H&E and Masson staining results are shown in Fig. 11. Different effects on bone regeneration were observed in all four groups. The best repair was achieved with the PGCL@5 %Ti_3_C_2_/5 %Van scaffold at either week 4 or week 8. The PGCL and PGCl@5 %Ti_3_C_2_ scaffolds did not contain antibiotics. Compared with that in the groups with Van, the local infection in the PGCL and PGCl@5 %Ti_3_C_2_ groups was aggravated, the inflammatory reaction around the scaffolds was obvious, and the morphology of the regenerated bone was irregular. The inflammatory response was not obvious in the PGCL@5 %Van group. However, owing to the lack of osteogenic activity, the bone repair effect was not as strong as that of the scaffold containing Ti_3_C_2_. The PGCL@5 %Ti_3_C_2_/5 %Van group presented a significant increase in the number and thickness of new bone. Compared with that in the PGCL@5 %Ti_3_C_2_/5 %Van group, the local inflammatory response in the PGCL@5 %Ti_3_C_2_/5 %Van group was reduced. The PGCL@5 %Ti_3_C_2_/5 %Van scaffold not only released antibiotics to inhibit bacteria and reverse the microenvironment of infection but also regulated the anti-inflammatory response through the use of Ti_3_C_2_ as an anti-inflammatory component [41]. The results of *in vivo* osteogenesis fully demonstrated that the PGCL@5 %Ti_3_C_2_/5 %Van scaffold has an outstanding ability to promote the repair of infected bone defects.

At the 4th week, many black Ti_3_C_2_ particles were present at the positions of the scaffolds in the PGCL@%Ti_3_C_2_ and PGCL@5 %Van/5 %Ti_3_C_2_ groups, indicating that the scaffolds had not been completely degraded. At the 8th week, the Ti_3_C_2_ particles were hardly visible at the position of the scaffold in the PGCL@5 %Van/5 %Ti_3_C_2_ group, suggesting that the scaffold had been basically degraded and that the Ti_3_C_2_ released during the degradation process of the scaffold had been metabolized. However, the PGCL@%Ti_3_C_2_ scaffold still contained many Ti_3_C_2_ particles, indicating that the degradation rate of the scaffold in the PGCL@%Ti_3_C_2_ group was slower than that of the scaffold in the PGCL@5 %Van/5 %Ti_3_C_2_ group. This result is essentially consistent with the *in vitro* degradation results of the scaffolds (Fig. 7A and C).

As shown in Fig. S5, H&E staining was performed on the heart, liver, spleen, lungs, and kidneys of rats from all groups to assess the *in vivo* safety of the material. The structures of the heart, liver, spleen, lungs, and kidneys in all groups of animals remained intact, with no significant abnormalities observed. These results indicate that the materials used in this study are safe for *in vivo* applications. As shown in Tables S3 and S4, the immune markers and blood chemistry of the rats were analysed to further investigate the safety of MXene materials for *in vivo* applications at 8 weeks post-operation. As shown in Table S3 and Table S3 S4, immune marker and blood chemistry analyses revealed that the PGCL@5 %Van/5 %Ti_3_C_2_ scaffold containing MXene materials did not exhibit significant hepatotoxicity, nephrotoxicity, immune response, or chronic inflammatory reactions compared with the Sham group after long-term *in vivo* implantation.

### 3.9 Molecular mechanism of osteogenesis and anti-inflammatory effects

**Fig. 12:**
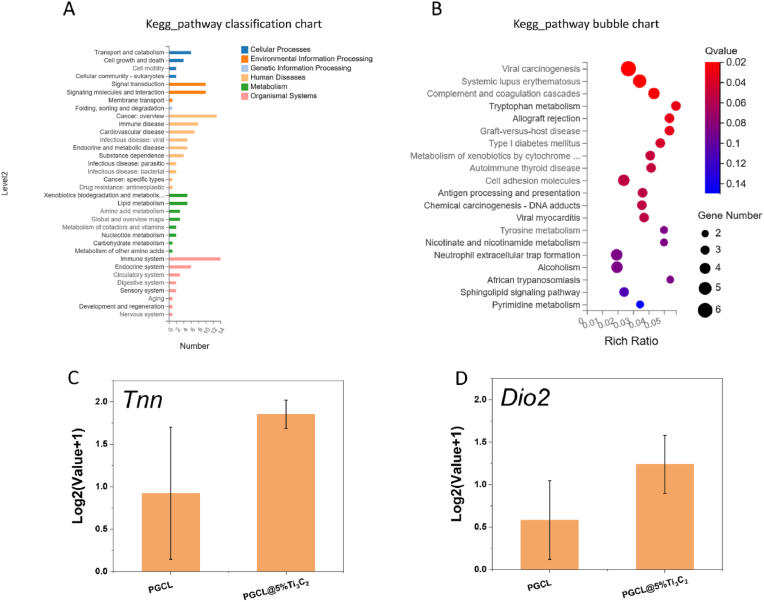
Analysis of the differentially expressed mRNAs in MC-3T3-E1 cells grown on PGCL and the PGCL@5 %Ti_3_C_2_ scaffolds for 14 days. (A) Bubble chart and (B) classification chart of differentially expressed genes in KEGG pathway. (C) and (D) Genes related to osteogenic differentiation among the differentially expressed genes, n = 3 ∗*P* < 0.05.

The differentially expressed mRNAs in MC-3T3-E1 cells grown on the PGCL@5 %Ti_3_C_2_ and PGCL scaffolds for 14 days were sequenced and analysed. The detailed information of all the differentially expressed mRNAs between PGCL and PGCL@5 %Ti_3_C_2_ is listed in Table S5 and Table S6, including 105 mRNAs, of which 60 genes were upregulated and 45 genes were downregulated. Fig. 12A shows the KEGG pathway classification chart, and Fig. 12B shows the KEGG pathway bubble chart. These differentially expressed genes were classified into transport and catabolism cellular processes. cell growth and death, environmental information processing, signal transduction, metabolism, signalling molecules and interaction organismal systems, membrane transport, immune system, development and regeneration. Some genes involved in the regulation of osteogenic signalling pathways were included among these DEGs. As shown in Fig. 12C, the expression of the tenascin N (Tnn) gene was upregulated in the PGCL@5 %Ti_3_C_2_ group. The Tnn gene is located in the nucleus and regulates basic cellular functions such as transcription, translation, proliferation, growth and survival when the PI3K-Akt pathway is activated by cell stimulation or toxic injury [39]. Takashi Fujita et al. reported that the binding of Runx2 to DNA can be enhanced by regulating the PI3K-Akt signalling pathway, thereby increasing the activity of Runx2 during osteoblast differentiation. As shown in Fig. S6 A-C, the results of IF indicated that the expression levels of the Runx2 and COL-I genes in the cells of the group containing MXene were significantly greater than those in the group without MXene. As a key transcription factor for osteogenic differentiation, Runx2 plays an important role in regulating the osteogenic differentiation of cells. Runx2 can directly regulate the expression of COL-I, which is the main component of the bone matrix. A large amount of COL-I deposition at the site of a bone defect can accelerate the process of bone mineralization [42]. As shown in Fig. 12D, the expression of the deiodinase iodothyronine type II (DiO2) gene was upregulated in the PGCL@5 %Ti_3_C_2_ group. DiO2 controls inflammation in bone tissue by downregulating the nuclear factor-κb (NF-κB) pathway [43]. IL-6 is a downstream target gene of NF-κB, and NF-κB directly induces IL-6 expression [44]. TNF-α acts as an upstream activator of NF-κB and is capable of directly activating the NF-κB pathway [45]. The high expression levels of IL-6 and TNF-α in cells indicate a severe inflammatory response in these tissues. As shown in Fig. 9A and B, the IF results indicated that the expression levels of IL-6 and TNF-α in the PGCL@5 %Ti_3_C_2_ group were lower than those in the PGCL group at the 1 W and 4 W time points. Quantitative mean fluorescence intensity results also showed that the expression levels of IL-6 and TNF-α in the PGCL@5 %Ti_3_C_2_ group were significantly lower than those in the PGCL group at the 1 W time point (∗*P* < 0.05) (Fig.S7 A and B). These results indicate that the scaffold containing Ti_3_C_2_ can reduce cellular inflammatory responses by regulating the NF-κB pathway.

## 4 Conclusion

In summary, a novel 3D-printed PGCL@Ti_3_C_2_ dual-function composite scaffold loaded with Van for acute infected bone defect regeneration was fabricated. The surface morphology, hydrophilicity, mechanical properties, degradation, mineralization, antibacterial ability, cytocompatibility and osteogenic differentiation ability of the composite scaffold were systematically investigated *in vitro*. The dual-function composite scaffold exhibited the best mineralization, antibacterial ability and osteogenic activity and satisfactory degradability and mechanical properties. The *in vivo* results showed that the PGCL@5 %Ti_3_C_2_/5 %Van scaffold effectively inhibited the infection caused by *MRSA* in bone defects and promoted bone repair. Transcriptome analysis revealed the potential mechanism of Ti_3_C_2_ in anti-inflammatory and osteogenic induction. Therefore, the PGCL@5 %Ti_3_C_2_/5 %Van scaffold can enhance the regeneration of acutely infected bone defects *in vivo*, indicating good clinical application prospects.

## CRediT authorship contribution statement

**Xipeng Chen:** Writing – review & editing, Writing – original draft, Methodology, Formal analysis, Data curation, Conceptualization. **Yuanpei Cheng:** Software, Methodology, Investigation, Data curation. **Yongbo Li:** Methodology, Formal analysis, Data curation. **Ze Tan:** Software, Project administration, Methodology, Formal analysis, Data curation. **Han Wu:** Writing – review & editing, Supervision, Project administration, Funding acquisition, Conceptualization.

## Declaration of competing interest

The authors declare that they have no known competing financial interests or personal relationships that could have appeared to influence the work reported in this paper.

## Data Availability

Data will be made available on request.
