# Supplementary material for: 3D-printed vancomycin-eluting PGCL/MXene bifunctional scaffold for management of infected bone defects
Source: Mater Today Bio. 2025 May 7;32:101847. doi: 10.1016/j.mtbio.2025.101847 (PMC12139433; doi:10.1016/j.mtbio.2025.101847)
Supplement: Multimedia component 1 [file mmc1.docx]

**Supplementary material**

**3D-printed vancomycin-eluting PGCL/MXene bifunctional scaffold for management of infected bone defects**

Xipeng Chen ^a^, Yuanpei Cheng ^a^, Yongbo Li ^b^, Ze Tan ^a^, Han Wu ^a,*^

*^a^* *Department of Orthopedics, China-Japan Union Hospital of Jilin University, Changchun, 130033, China*

*^b^ Shandong First Medical University Affiliated Provincial Hospital, Jinan, 250100, China*

* Correspondence to: Han Wu, Department of Orthopedics, China-Japan Union Hospital of Jilin University, Changchun, 130033, China.

E-mail addresses: wu_han@jlu.edu.cn


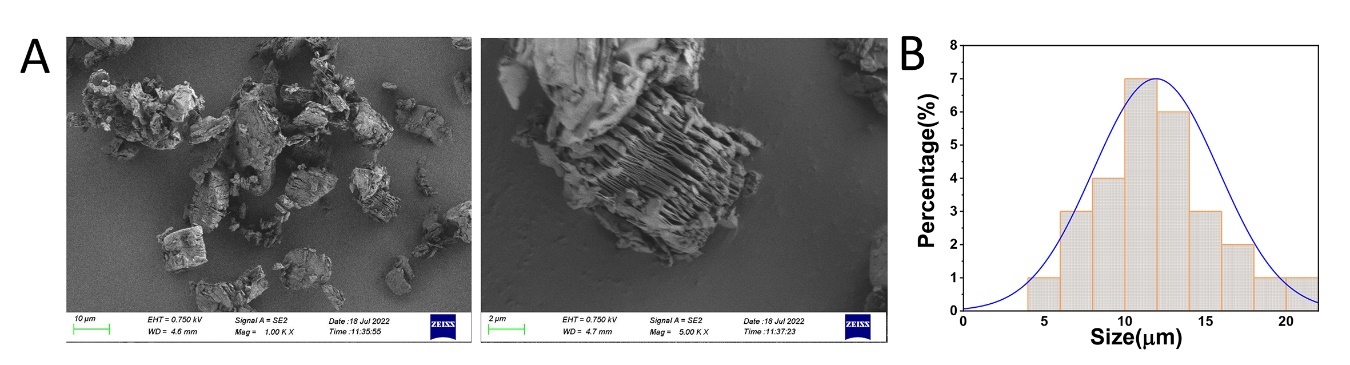


Fig.S1. (A) Ti_3_C_2_ particles SEM images, (B) particle size distribution of Ti_3_C_2_.


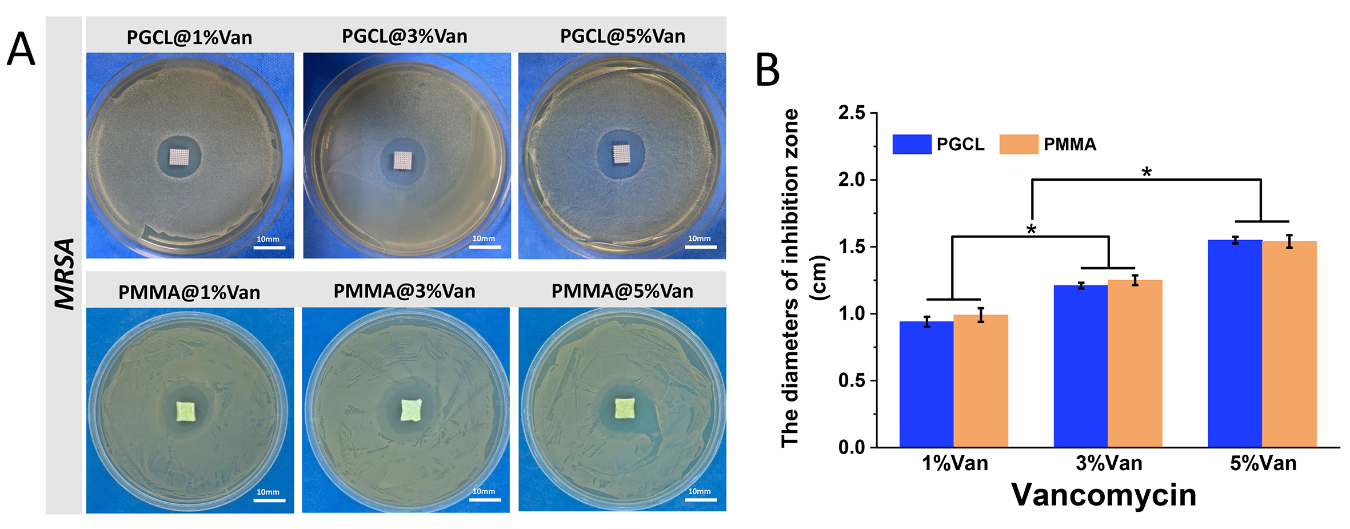


Fig.S2. (A) Images of inhibition zone and (B) the diameters of inhibition zone for PMMA cement blocks and PGCL scaffolds with different proportions of Van, n=3 **P*<0.05.


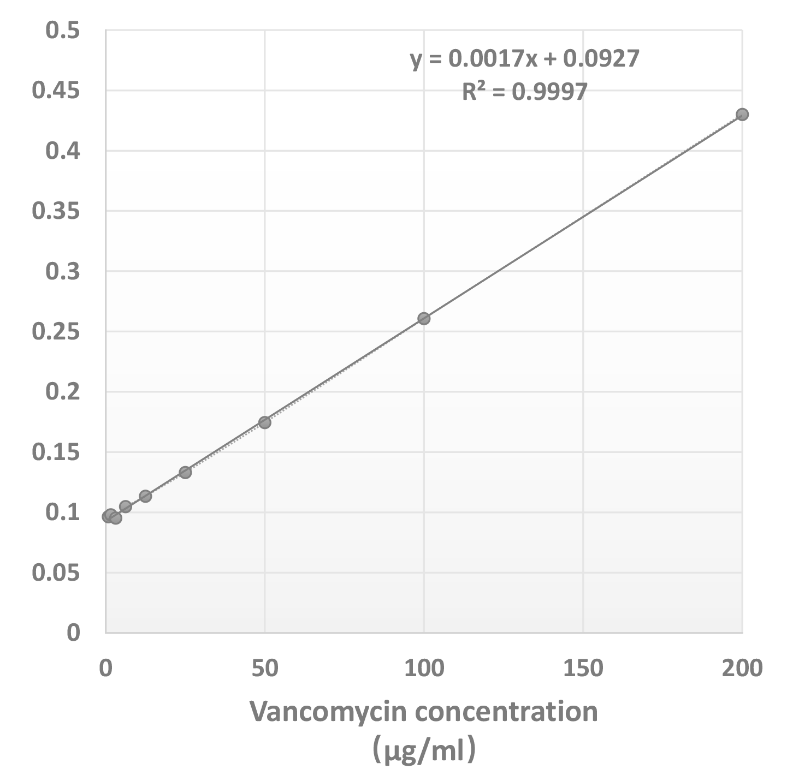


Fig.S3. Standard curve of different concentrations of van versus OD280 values.


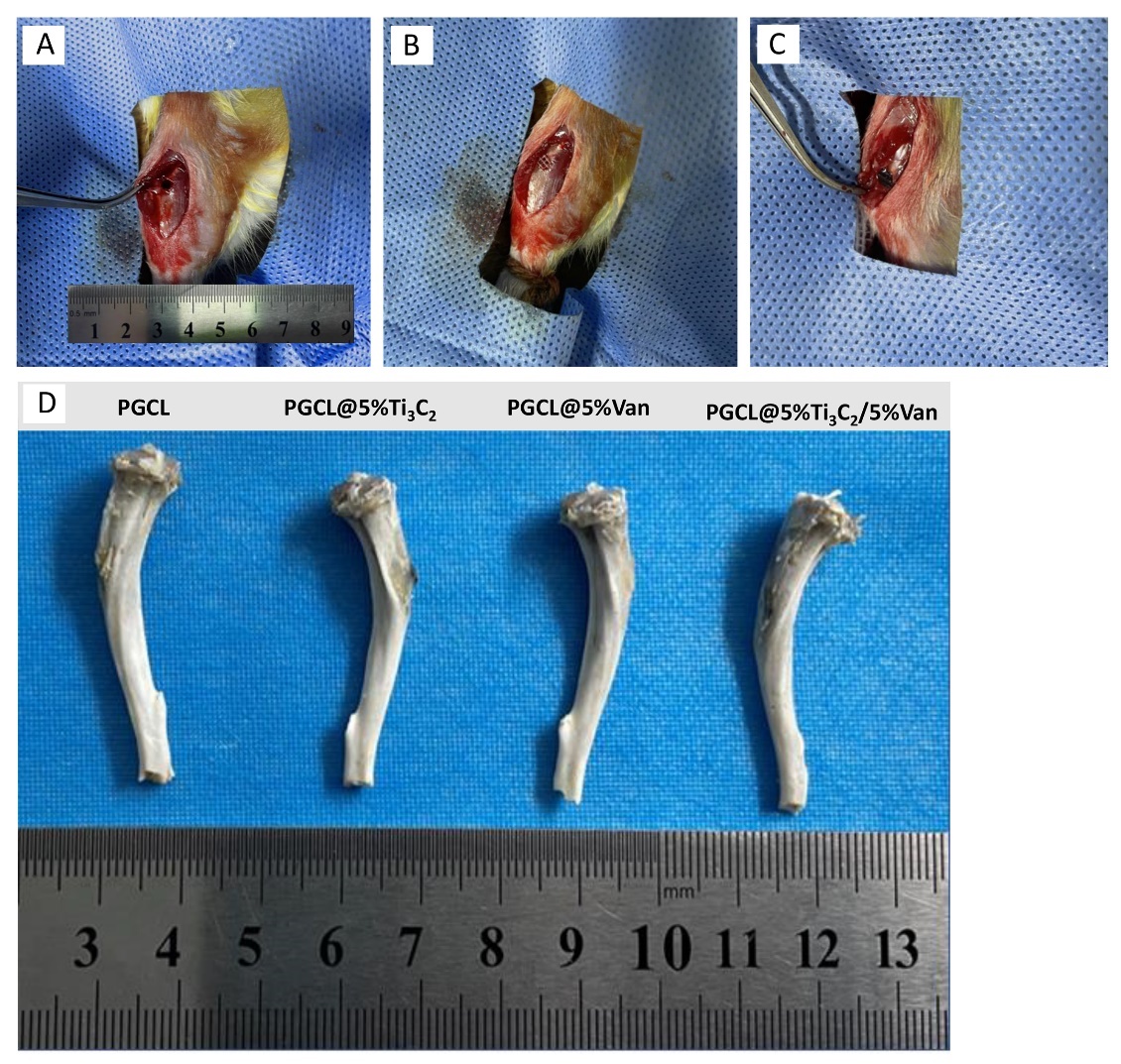


Fig.S4. (A-C) The process of establishing a rat tibial bone defect model and implanting the scaffold. (D) The appearance of the repaired rat tibia.


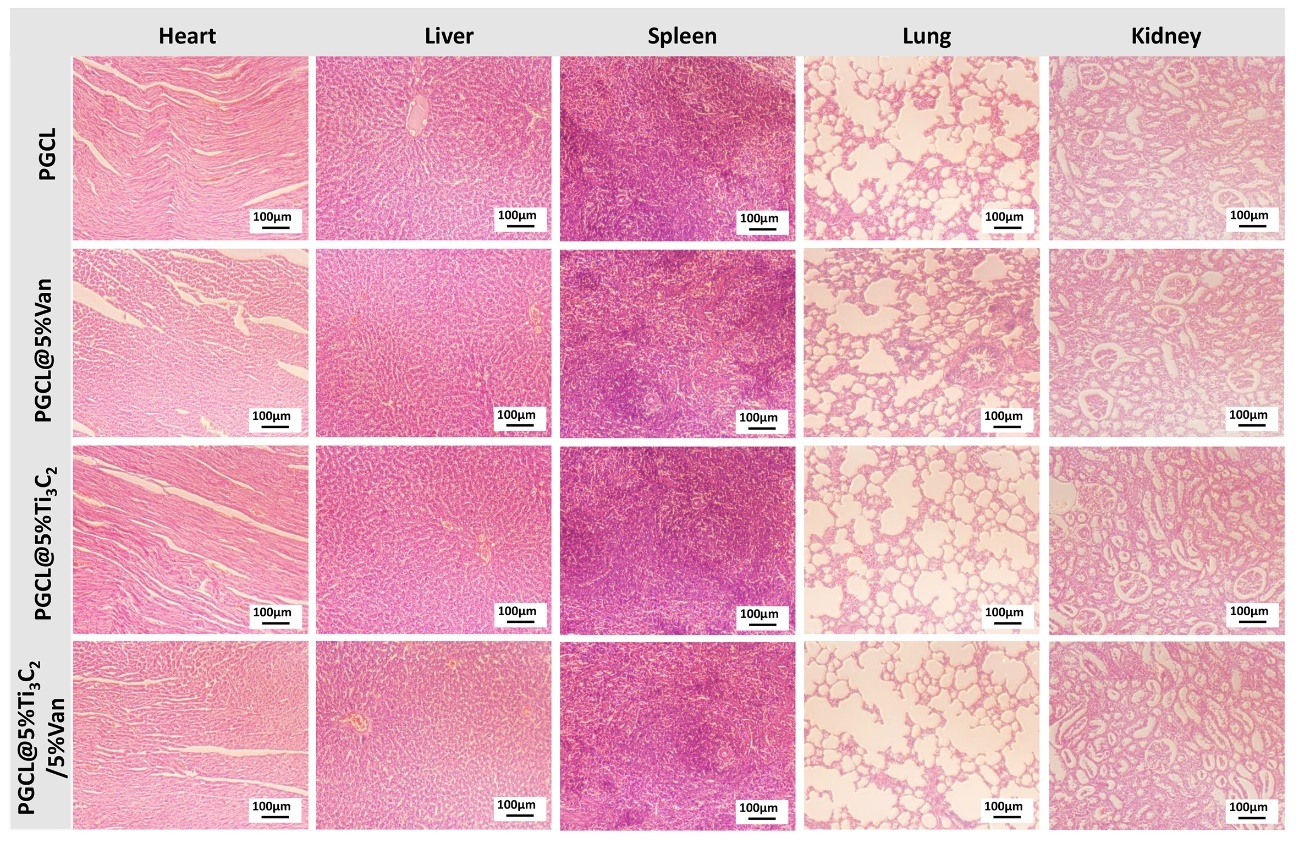


Fig.S5. H&E images of the heart, liver, spleen, lungs, and kidneys of rats.


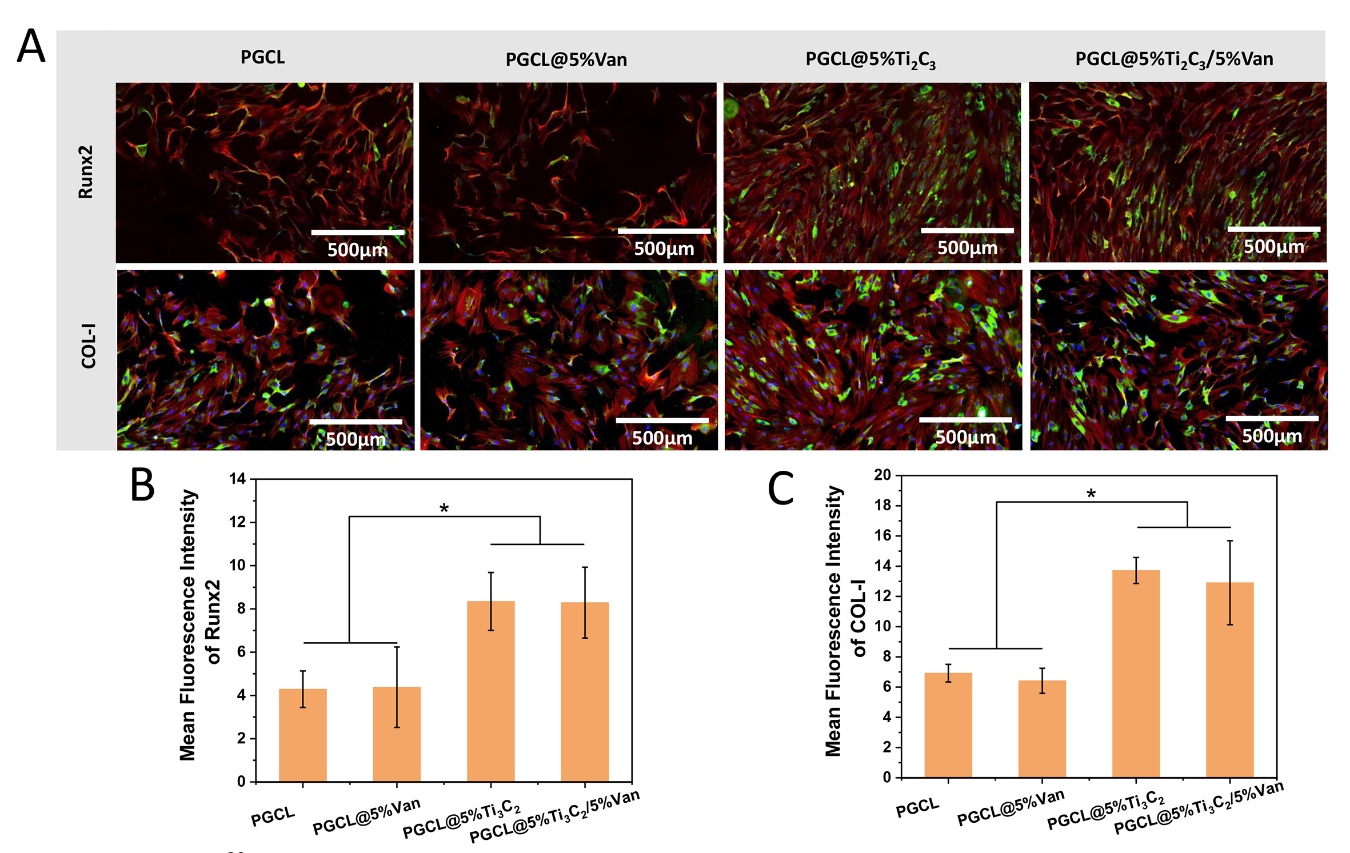


Fig.S6. (A) IF images of Runx2 and COL-I in MC-3T3-E1 cells, (B) mean fluorescence intensity of Runx2 and (C) COL-I in IF images, **P*<0.05.


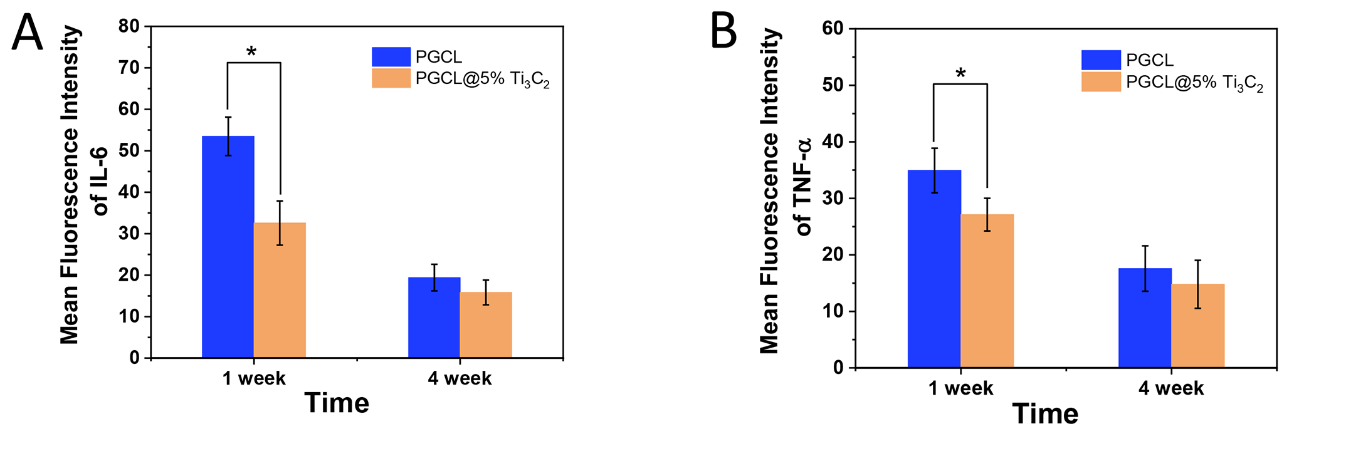


Fig.S7. (A) The mean fluorescence intensity of IF images for (A) IL-6 and (B) TNF-α at 1 week and 4 weeks after scaffold implantation in rats.

**Table S1. Weight percentages of PGCL and Ti_3_C_2_ or** **Van in different groups**.

| Group name | PCL weight percentage (%) | Ti_3_C_2_ weight percentage (%) | Van weight percentage (%) |
| --- | --- | --- | --- |
| PGCL | 100% |  |  |
| PGCL@1%Ti_3_C_2_ | 99% | 1% |  |
| PGCL@5%Ti_3_C_2_ | 95% | 5% |  |
| PGCL@10%Ti_3_C_2_ | 90% | 10% |  |
| PGCL@1%Van | 99% |  | 1% |
| PGCL@3%Van | 97% |  | 3% |
| PGCL@5%Van | 95% |  | 5% |
| PGCL@10%Van | 10% |  | 10% |
| PGCL@5%Ti_3_C_2_/5%Van | 90% | 5% | 5% |

**Table S2. The number of leukocytes in the serum of rat post-surgery.**

| Groups | 3 Days | 7 Days |
| --- | --- | --- |
|  | 10^9^/L | |
| Blank | 13.55±1.32 | 19.80±2.91 |
| PGCL | 13.32±1.79 | 15.51±0.89 |
| PGCL@5%Ti_3_C_2_ | 11.55±1.25 | 15.17±2.57 |
| PGCL@5%Van | 8.43±1.00 | 9.42±0.83 |
| PGCL@ 5%Ti_3_C_2_/5%Van | 8.39±0.73 | 8.76±1.13 |

**Table S3. Blood routine analysis at 8w after scaffold implantation.**

| Name | Sham | PGCL@ 5%Ti_3_C_2_/5%Van |
| --- | --- | --- |
| ALT (U/L) | 32.6±2.46 | 37.9±2.35 |
| AST (U/L) | 99.3±4.34 | 96.5±3.76 |
| LDH (U/L) | 626.2±19.36 | 641.9±24.55 |
| UA (μmol/L) | 75.2±4.64 | 78.5±3.98 |
| CREA (μmol/L) | 28.0±1.22 | 27.8±1.41 |
| ALB (g/L） | 33.0±1.20 | 31.0±1.50 |
| ALP (U/L) | 265.2±12.43 | 285.3±16.43 |
| UREA (mmol/L) | 6.8±0.78 | 6.9±0.54 |
| TP (g/L) | 58.50±3.54 | 55.85±5.32 |

**Table S4. Infection and immune system analysis.**

| Name | Sham | PGCL@ 5%Ti_3_C_2_/5%Van |
| --- | --- | --- |
| IL-1β (pg/mL) | ＜2.5 | ＜2.5 |
| IL-2 pg/mL) | ＜2.5 | ＜2.5 |
| IL-6 (pg/mL) | ＜2 | ＜2 |
| IL-8 (pg/mL) | 93.1±1.51 | 89.4±1.62 |
| TNF-α (pg/mL) | 10.7±0.55 | 9.6±0.91 |
| IgG (g/L) | 0.22±0.021 | 0.24±0.035 |
| IgA (g/L) | 0.034±0.0031 | 0.038±0.0040 |
| IgM (g/L) | 0.02±0.006 | 0.03±0.012 |
| IgE (IU/mL) | 6.61±0.252 | 6.25±0.195 |
| C3 (g/L) | 0.11±0.006 | 0.10±0.006 |
| C4 (g/L) | 0.006±0.0006 | 0.007±0.0006 |

**Table S5. Upregulated genes identified in transcriptomic analysis.**

| Gene ID | Gene Symbol | Type | log2 (PM/P) | Qvalue (PM/P) |
| --- | --- | --- | --- | --- |
| 100039324 | 'Gm10147' | mRNA | 1.595311 | 1.57E-17 |
| 100039377 | 'Gm10096' | mRNA | 7.586966 | 2.93E-21 |
| 100039890 | 'Gm15093' | mRNA | 2.249569 | 1.48E-07 |
| 100042807 | 'Eif3j2' | mRNA | 1.71001 | 3.99E-04 |
| 100504195 | 'Micalcl' | mRNA | 1.146232 | 4.98E-06 |
| 105244828 | 'Gm40364' | mRNA | 4.782535 | 5.28E-04 |
| 105244829 | 'Gm40365' | mRNA | 4.6246 | 4.05E-05 |
| 105245342 | 'Gm40814' | mRNA | 6.488901 | 1.76E-09 |
| 105980076 | 'Gm45929' | mRNA | 5.44465 | 6.20E-06 |
| 11303 | 'Abca1' | mRNA | 1.283372 | 2.15E-22 |
| 115487414 | 'Gm51777' | mRNA | 4.792185 | 5.01E-04 |
| 115488169 | 'Gm52051' | mRNA | 1.315415 | 5.49E-05 |
| 11606 | 'Agt' | mRNA | 1.296319 | 4.68E-21 |
| 11688 | 'Alox8' | mRNA | 1.051455 | 8.01E-04 |
| 117167 | 'Steap4' | mRNA | 1.093881 | 2.10E-05 |
| 118567557 | 'LOC118567557' | mRNA | 1.177934 | 6.26E-06 |
| 118568144 | 'LOC118568144' | mRNA | 4.70178 | 8.10E-04 |
| 118568301 | 'LOC118568301' | mRNA | 1.380105 | 6.22E-04 |
| 12268 | 'C4b' | mRNA | 1.041043 | 1.08E-46 |
| 12489 | 'Cd33' | mRNA | 1.046715 | 5.99E-04 |
| 12515 | 'Cd69' | mRNA | 1.387795 | 4.03E-04 |
| 12516 | 'Cd7' | mRNA | 2.359225 | 8.39E-05 |
| 13078 | 'Cyp1b1' | mRNA | 1.117998 | 7.71E-46 |
| 13371 | 'Dio2' | mRNA | 1.106192 | 2.33E-09 |
| 14204 | 'Il4i1' | mRNA | 2.013625 | 6.05E-04 |
| 15439 | 'Hp' | mRNA | 1.274337 | 1.50E-04 |
| 15483 | 'Hsd11b1' | mRNA | 1.844652 | 6.82E-05 |
| 16644 | 'Kng1' | mRNA | 1.656956 | 4.56E-05 |
| 17381 | 'Mmp12' | mRNA | 1.187857 | 2.24E-06 |
| 17476 | 'Mpeg1' | mRNA | 1.024358 | 3.20E-05 |
| 18429 | 'Oxt' | mRNA | 1.501868 | 7.48E-04 |
| 19142 | 'Prss12' | mRNA | 1.10601 | 1.07E-04 |
| 20210 | 'Saa3' | mRNA | 1.022798 | 2.11E-31 |
| 20704 | 'Serpina1e' | mRNA | 1.38789 | 3.55E-05 |
| 20716 | 'Serpina3n' | mRNA | 1.870279 | 4.44E-14 |
| 208164 | 'Fam180a' | mRNA | 1.593691 | 9.04E-05 |
| 209588 | 'Sectm1a' | mRNA | 1.496729 | 1.05E-44 |
| 22526 | 'Gm4836' | mRNA | 1.14354 | 4.16E-11 |
| 233752 | 'Insc' | mRNA | 1.287276 | 7.87E-06 |
| 319155 | 'H4c3' | mRNA | 2.249958 | 2.97E-05 |
| 329278 | 'Tnn' | mRNA | 1.026242 | 5.73E-11 |
| 329436 | 'Gm14461' | mRNA | 1.747791 | 1.72E-04 |
| 382277 | 'Gm5169' | mRNA | 1.761047 | 8.14E-04 |
| 385643 | 'Kng2' | mRNA | 1.060017 | 4.01E-04 |
| 545649 | 'Gm13276' | mRNA | 1.249551 | 2.48E-07 |
| 626578 | 'Gbp10' | mRNA | 2.756272 | 1.77E-04 |
| 665596 | 'H2bc23' | mRNA | 1.065945 | 9.79E-35 |
| 666806 | 'Gm8300' | mRNA | 1.379265 | 6.08E-04 |
| 667034 | 'Pnp2' | mRNA | 1.227734 | 6.57E-07 |
| 67374 | 'Jam2' | mRNA | 1.707149 | 6.74E-04 |
| 68957 | 'Paqr6' | mRNA | 1.774263 | 1.66E-04 |
| 69824 | 'Glod5' | mRNA | 1.54176 | 1.10E-05 |
| 70574 | 'Cpm' | mRNA | 1.257434 | 2.64E-06 |
| 71724 | 'Aox3' | mRNA | 1.481199 | 3.53E-19 |
| 72003 | 'Synpr' | mRNA | 1.52915 | 3.49E-04 |
| 75668 | 'Rasl10a' | mRNA | 1.020424 | 3.41E-04 |
| 75767 | 'Rab11fip1' | mRNA | 1.325148 | 1.54E-04 |
| 76166 | 'Cplane2' | mRNA | 1.501966 | 8.72E-06 |
| 93703 | 'Pcdhgb6' | mRNA | 2.465534 | 1.17E-04 |
| 93710 | 'Pcdhga2' | mRNA | 2.327376 | 1.66E-10 |

**Table S6. Downregulated genes identified in transcriptomic analysis.**

| Gene ID | Gene Symbol | Type | log2 (PM/P) | Qvalue (PM/P) |
| --- | --- | --- | --- | --- |
| 100039467 | 'Gm10487' | mRNA | -1.48626 | 2.62E-09 |
| 100042074 | 'Gm3650' | mRNA | -1.65205 | 1.25E-13 |
| 100042109 | 'Gm10488' | mRNA | -2.05514 | 5.39E-32 |
| 100042144 | 'Gm14632' | mRNA | -11.9954 | 9.32E-06 |
| 100042175 | 'Gm10230' | mRNA | -1.06476 | 2.61E-09 |
| 100042295 | 'Gm3776' | mRNA | -1.65478 | 1.84E-04 |
| 102635181 | 'Gm32584' | mRNA | -2.12564 | 2.78E-12 |
| 102902673 | 'Gm21992' | mRNA | -4.98507 | 1.33E-04 |
| 108167548 | 'Gm45855' | mRNA | -1.35115 | 1.59E-04 |
| 108168114 | 'Gm5451' | mRNA | -1.70757 | 5.82E-05 |
| 110558 | 'H2-Q9' | mRNA | -3.38772 | 1.29E-04 |
| 11576 | 'Afp' | mRNA | -1.57276 | 2.03E-09 |
| 118567440 | 'LOC118567440' | mRNA | -4.7293 | 5.86E-04 |
| 14997 | 'H2-M9' | mRNA | -1.1554 | 1.79E-12 |
| 15112 | 'Hao1' | mRNA | -1.06704 | 2.16E-04 |
| 17287 | 'Mep1a' | mRNA | -1.26038 | 4.95E-04 |
| 17384 | 'Mmp10' | mRNA | -1.55097 | 1.60E-07 |
| 18102 | 'Nme1' | mRNA | -1.00026 | 7.32E-9 |
| 8811 | 'Prl2c2' | mRNA | -1.17975 | 4.32E-5 |
| 18812 | 'Prl2c3' | mRNA | -1.89104 | 5.69E-6 |
| 20609 | 'Sstr5' | mRNA | -1.78867 | 8.67E-08 |
| 213948 | 'Atg9b' | mRNA | -1.11043 | 6.52E-11 |
| 21956 | 'Tnnt2' | mRNA | -2.79278 | 1.35E-10 |
| 224079 | 'Atp13a4' | mRNA | -3.21921 | 6.54E-06 |
| 224756 | 'H2-M1' | mRNA | -1.42312 | 8.20E-88 |
| 243529 | 'H1f10' | mRNA | -1.35331 | 1.38E-93 |
| 319168 | 'H2ac12' | mRNA | -4.74721 | 5.33E-04 |
| 319259 | 'Bricd5' | mRNA | -2.54929 | 1.35E-04 |
| 319482 | '9530053A07Rik' | mRNA | -1.30878 | 1.09E-04 |
| 331195 | 'Pramel39' | mRNA | -4.65535 | 8.67E-04 |
| 53972 | 'Ngef' | mRNA | -1.56134 | 6.43E-24 |
| 545651 | 'Gm13278' | mRNA | -1.44813 | 2.16E-10 |
| 56089 | 'Ramp3' | mRNA | -1.21646 | 5.17E-04 |
| 56460 | 'Pkp3' | mRNA | -1.04071 | 7.19E-04 |
| 620499 | 'Gm6158' | mRNA | -6.17397 | 1.55E-6 |
| 664968 | 'Tmem238' | mRNA | -1.02387 | 7.32E-11 |
| 665622 | 'H2bc24' | mRNA | -3.88717 | 5.31E-87 |
| 667962 | 'Zfp966' | mRNA | -1.25356 | 2.65E-09 |
| 72544 | 'Exosc6' | mRNA | -3.77041 | 3.75E-49 |
| 74626 | 'Tmem81' | mRNA | -3.15135 | 1.54E-15 |
| 78444 | 'Pgpep1l' | mRNA | -1.35614 | 3.11E-43 |
| 93712 | 'Pcdhga4' | mRNA | -1.00477 | 1.23E-04 |
| 93717 | 'Pcdhga9' | mRNA | -1.56738 | 7.03E-06 |
| 93898 | 'Cers1' | mRNA | -5.33867 | 1.43E-27 |
| 94180 | 'Acsbg1' | mRNA | -1.09918 | 2.30E-36 |
